# Effect of Chain Stereoconfiguration on Poly(3-hydroxybutyrate) Crystallization Kinetics

**DOI:** 10.1021/acs.biomac.2c00682

**Published:** 2022-08-05

**Authors:** Maria Rosaria Caputo, Xiaoyan Tang, Andrea H. Westlie, Haritz Sardon, Eugene Y.-X. Chen, Alejandro J. Müller

**Affiliations:** † POLYMAT and Department of Polymers and Advanced Materials: Physics, Chemistry and Technology, Faculty of Chemistry, University of the Basque Country UPV/EHU, Paseo Manuel de Lardizabal 3, 20018 Donostia-San Sebastián, Spain; ‡ Department of Chemistry, Colorado State University, Fort Collins, Colorado 80523-1872, United States; § IKERBASQUE, Basque Foundation for Science, Plaza Euskadi 5, 48009 Bilbao, Spain

## Abstract

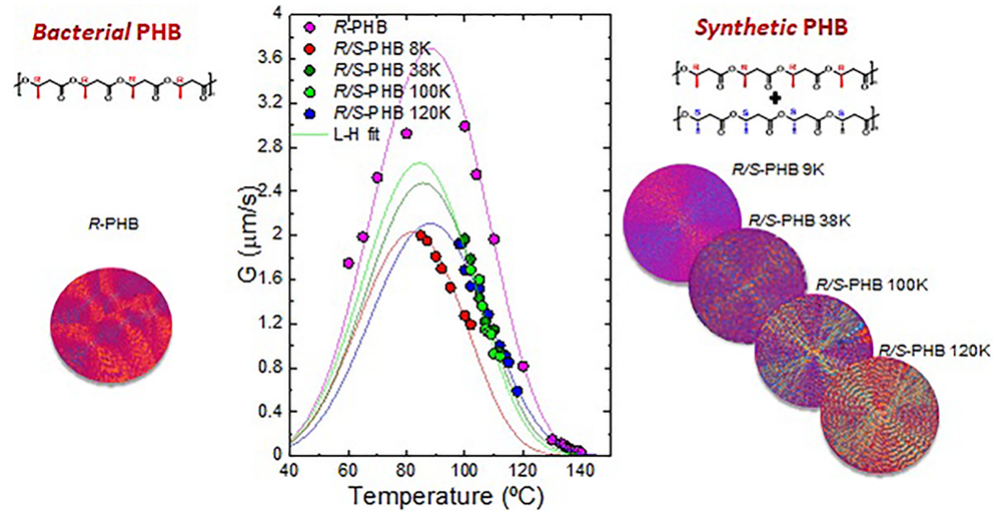

Poly(3-hydroxybutyrate) (PHB) is naturally accumulated by bacteria but can also be synthesized chemically. Its processability is limited, as it tends to degrade at temperatures above its melting temperature; hence, investigation into crystallization kinetics and morphology of PHB materials of both natural and synthetic origins is of great need and interest to get a better understanding of structure–property relationship. Accordingly, this contribution reports a first study of the crystallization and morphology of synthetic PHB materials of different molecular weights. These synthetic PHBs are racemic mixtures (50/50 mol %) of *R* and *S* chain configurations and are compared with an enantiopure bacterial *R*-PHB. Nonisothermal and isothermal crystallization studies show that *R* and *S* chains of PHB can cocrystallize in the same unit cell as the *R*-PHB. Most significantly, the results show that the presence of *S* chains decreases the overall crystallization rate, which could enhance the processability and industrialization of PHB-based materials.

## 1 Introduction

Over the past few decades, the massive use of petroleum-based plastics, polyolefins, in particular, has led to an increase in problems related to their disposal. Recycling these materials to obtain value-added products^1−6^ has been one of the main solutions implemented to manage the environmental problem associated with plastic disposal. Additionally, major R&D efforts are directed at developing biobased and biodegradable polymers that are characterized by an intrinsically lower environmental impact.^7,8^

In this context, biobased polyesters, particularly polyhydroxyalkanoates (PHAs), are more environmentally benign, sustainable plastics. PHAs were discovered by Leimogne in 1925, in the form of poly(3-hydroxybutyrate) (PHB) in a bacterium called *Bacillus megaterium*.^9^ Natural PHAs are produced by various microorganisms that can store them in their cytoplasm as a source of energy.^10,11^ Bacteria can store PHAs as granules in large amounts: the amount of polyesters reaches up to 90% by weight of the dry cells. The most important aspect of this class of materials is that PHAs are 100% of biological origin and can degrade under different environmental conditions.^12^ These features make PHAs fall into the group of materials that can be defined as both “biobased” and “biodegradable”, according to the ASTM D6866 and ASTM D6400.

**Scheme 1:**
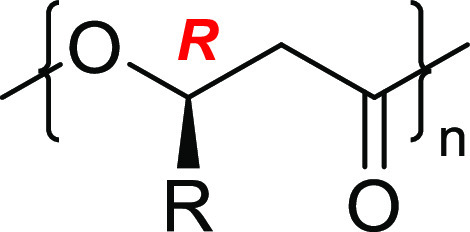
General Structure of PHAs

From a chemical point of view, bacterial PHAs are optically active polyesters^12,13^ due to their absolute (*R*) main-chain chirality, and their general structure is given in Scheme 1.

**Scheme 2:**
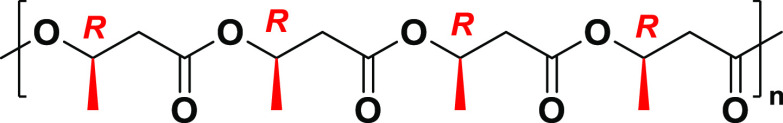
Structure of Poly[(*R*)-3-hydroxybutyrate] (*R*-PHB)

The pendant group *R* can have different configurations with respect to the main-chain backbone, leading to different stereochemically defined PHAs with different properties. The most studied is PHB, the simplest yet most important member of the large PHA family; the purely isotactic PHB is a polymer whose thermal properties are comparable to those of isotactic polypropylene. As other PHAs, the bacterial PHB is enantiomerically pure, with stereocenters being *R* configuration only, and its structure is represented in Scheme 2.

PHB has good resistance to moisture, excellent barrier to gas, insolubility in water, and also some resistance to hydrolytic degradation and ultraviolet rays.^12,14−16^ However, it also has several drawbacks: it is brittle and suffers from thermal decomposition at temperatures just above its melting temperature (*T*_m_). These problems can be solved, in part, by producing blends with other polyesters or by copolymerizing it with suitable comonomers.^17^ Owing to these drawbacks, PHB has not been widely employed industrially, as it is difficult to process and mechanically brittle. Additionally, its biosynthesis (by bacteria) is slow, as it is the process of extracting it from bacteria. As a result, its cost is high in comparison with commodity plastics. Furthermore, as biosynthesis is a polycondensation process in nature, it is difficult to control the resulting PHB molecular weight and dispersity. For these reasons, developing the chemical synthesis of PHA homopolymers and copolymers with controlled molecular weight and low dispersity values as well as diverse stereomicrostructures is highly desirable.

**Scheme 3:**
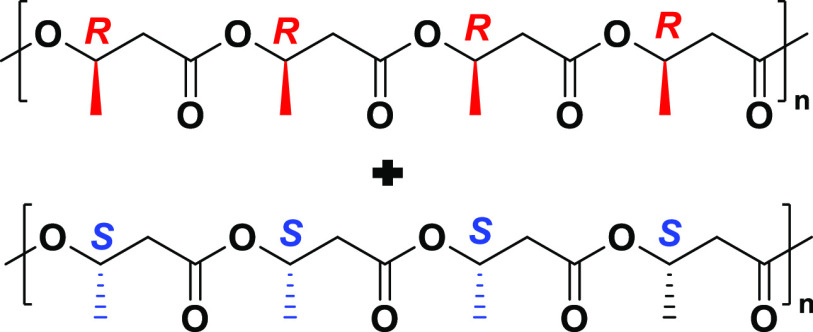
Schematic Structure of Synthetic Racemic, Isotactic PHB Consisting of *R* and *S* Chains in a 50/50 Composition

Two main synthetic routes have been developed, both based on the ring-opening polymerization (ROP) of cyclic esters. The four-membered ring *rac*-β-butyrolactone (β-BL) can be polymerized to iso-enriched, syndiotactic, or atactic P3HB, depending on the catalyst employed.^18^ Early organometallic catalyzed ROP of *rac*-β-BL by alkylaluminum species yielded mixtures of P3HB that could be fractionated into soluble atactic and insoluble isotactic fractions.^19−26^ These catalysts were often sluggish, and the low activity did not yield high molecular weight P3HB. Employment of discrete chromium salophen species in ROP of *rac*-β-BL showed much higher activity and allowed for the production of higher molecular weight P3HB but with broad dispersity (*Đ* ≥ 5.2) and reasonable isoselectivity (*P*_m_ up to 0.66).^27^ A discrete diiminate zinc alkoxide initiator was found to have very high activity resulting in controlled and high molecular weight with narrow dispersity, but atactic P3HB.^28^ The ROP of *rac*-β-BL with discrete yttrium complexes supported by tetradentate, dianionic alkyoxy-amino-bis(phenolate) ligands results in high activity, controlled reactivity, and high syndioselectivity.^29−31^ Recently, a new route based on the ROP eight-membered cyclic dimer of 3-hydroxybutyrate, or eight-membered dimethyl diolide (8DL^Me^) with two stereogenic centers, has been developed for the living synthesis of perfectly isotactic, syndiotactic, or stereodiblock P3HB materials.^32,33^ The use of discrete yttrium amido complexes supported by salcy ligands results was found to be highly stereoselective and highly active −100% conv. in <10 min to high molecular weight and narrow dispersity (*M*_n_ = 154 kg mol^–, 1^, *Đ* = 1.01) polymer.^33^ The isotactic polymer produced by the ROP of *rac*-8DL^Me^ was different than the biological PHB as the resulting PHB is a racemic mixture of chains with 50% *R* configuration and 50% *S* configuration represented in Scheme 3 below.

The crystallization kinetics of these novel 50/50 *R*/*S*-PHB materials have never been investigated and compared to the microbially derived *R*-PHB. To study the crystallization, morphology, and thermal properties of the synthetic *R*/*S*-PHBs (with different molecular weights), a comparative study with a commercial bacterial *R*-PHB has been performed for the first time. Although the crystalline structures of *R*/*S*-PHB and *R*-PHB are shown to be the same, the racemic mixture with both enantiomeric chain configurations is found to slow down the primary nucleation and the growth of the chain ensemble during crystallization in comparison with *R*-PHB chains.

## 2 Materials and Methods

Five different PHBs were used in this study: a bacterial PHB (denoted herein as *R*-PHB) obtained commercially in white powder form (without any additives), supplied by Sigma-Aldrich, and four laboratory synthesized PHBs (denoted as *R*/*S*-PHB in view of their 50/50 racemic mixture characteristics) prepared following the recently reported ROP eight-membered cyclic dimer of 3-hydroxybutyrate or eight-membered dimethyl diolide.^32,33^ The values of molecular weight and the dispersity for all the samples used in this work are reported in Table 1. The different synthetic PHBs are named based on their closest *M*_n_ value.

**Table 1:** Number (*M*_n_) and Weight (*M*_w_) Average Molecular Weight and the Dispersity (*Đ*) Values for the Bacterial and Synthetic PHB Materials Employed in This Study

sample	*M*_n_^a^ (g/mol)	*M*_w_^a^ (g/mol)	*Đ*
*R*-PHB	241000	633000	2.62
*R*/*S*-PHB 9 K	9000	9180	1.02
*R*/*S*-PHB 38 K	38000	40700	1.07
*R*/*S*-PHB 100 K	99000	103000	1.04
*R*/*S*-PHB 120 K	119000	133000	1.12

a Measured by GPC, as described by Tang et al.^33^

### 2.1 Characterization Methods

To eliminate residual moisture present in the polymer, which would contribute to degradation of the materials, the samples were placed in an oven for 24 h at 60 °C under vacuum before using them.

#### 2.1.1 NMR Spectroscopy

^1^H NMR spectra were recorded in a Bruker Avance DPX 300 at 300.16 MHz of resonance frequency. Samples of 10 mg were dissolved in deuterated chloroform (CDCl_3_) and heated for a few minutes at 60 °C to improve the dissolution. The experimental conditions were as follows: 3 s acquisition time; 1 s delay time; 8.5 μs pulse; spectral width 5000 Hz; and 32 scans.

#### 2.1.2 Termogravimetric Analysis

A PerkinElmer TGA was used to determine the temperature at which the samples thermally degrade in air. To perform this experiment, 10 mg of each sample were placed in a platinum crucible, and the following heat treatment was applied: heating from 30 to 500 °C at 20 °C/min.

#### 2.1.3 Differencial Scanning Calorimetric Analysis (DSC)

A PerkinElmer Pyris I DSC equipped with an Intracooler 2P was employed to characterize thermal properties. All the experiments were performed under ultrapure nitrogen flow, and the instrument was calibrated with indium and tin standards. Samples of 2 mg for each type of PHB were used. Measurements were performed by placing the samples in sealed aluminum pans.

Nonisothermal experiments of bacterial and synthetic PHBs were carried out following the same thermal protocol. The samples were first heated at 20 °C/min up to 190 °C and left at this temperature for 1 min to erase the thermal history, then, they were cooled to 20 °C/min down to −40 °C and held 1 min at this temperature. Finally, they were reheated at 20 °C/min up to 190 °C.

The thermal protocol used in the nonisothermal experiments was used to determine the thermal stability of both polymers. This protocol was applied to samples of 2 mg for each polymer and repeated 10 times. It was thus possible to determine the melting temperature, the crystallization temperature, and the relative enthalpies for each cycle number.

Moreover, to investigate the overall crystallization kinetics, an isothermal protocol was applied. First, the minimum isothermal crystallization temperature *T*_c,min_ was determined by trial and error following Lorenzo et al.^34^ Samples were cooled from the melt to *T*_c_ values (estimated from the nonisothermal DSC runs) at 60 °C/min and then immediately reheated at 20 °C/min up to 185 °C. This protocol was repeated cyclically, at decreasing *T*_c_, until no melting enthalpy was found in the reheating scan. After the *T*_c_ range was determined, the isothermal crystallization experiments were performed, closely following the procedure suggested by Lorenzo et al.:^34^ (I) heating from room temperature to 20 °C above the melting point at 20 °C/min, that is in our case 185 °C; (II) holding the sample for 1 min at that temperature to erase thermal history; (III) quenching the sample to a predetermined crystallization temperature (*T*_c_) at 60 °C/min; (IV) isothermal crystallization until maximum saturation (in our case 30 min for each *T*_c_); (V) heating from *T*_c_ to 185 °C at 20 °C/min to record the melting behavior after the isothermal crystallization. This final melting run provided the values of apparent melting points that were employed to perform the Hoffman–Weeks extrapolation to calculate the equilibrium melting temperature of each material. These experiments were performed on as-prepared samples, which were in powder form.

In addition, to obtain reproducible results, given the rapid degradation that PHB typically experiences upon melting, a different sample was used for each isothermal crystallization experiment.

#### 2.1.4 Wide-Angle X-ray Scattering

X-ray powder diffraction patterns at room temperature were collected by using a Philips X’pert PRO automatic diffractometer operating at 40 kV and 40 mA, in theta–theta configuration, secondary monochromator with Cu-Kα radiation (λ = 1.5418 Å) and a PIXcel solid-state detector (active length in 2θ 3.347°). Data were collected from 5° to 50° 2θ (step size = 0.026 and time per step = 60 s) at room temperature.

Furthermore, the X-ray diffraction profiles during the crystallization and melting process were collected following the procedure and conditions of nonisothermal experiments conducted in the DSC equipment. Wide-angle X-ray scattering (WAXS) experiments were measured at beamline BL11-NCD in the ALBA Synchrotron (Barcelona, Spain). Aluminum pans were employed to place samples in the beam path. A THMS600 Linkam hot stage and a liquid nitrogen cooling device were employed for temperature control and to heat and cool the samples. The X-ray energy source amounted to 12.4 keV. For WAXS, the sample–detector distance was 132.6 mm with a 21.2° tilt angle, and chromium(III) oxide was employed to do the calibration (Rayonix LX255-HS detector, Evanston, IL, U.S.A., with a resolution of 1920 × 5760 pixels and pixel size of 44 μm^2^).

#### 2.1.5 Polarized Light Optical Microscope Analysis

A polarized light optical microscope, Olympus BX51 (Olympus, Tokyo, Japan), equipped with an Olympus SC50 digital camera and with a Linkam-15 TP-91 hot stage (Linkam, Tadworth, U.K.; coupled to a liquid nitrogen cooling system) was used to observe the morphology of the samples, after crystallization from the melt. Films with around 100 μm thickness were prepared by melting the samples between two glass slides. The samples were heated to 190 °C to erase their thermal history, kept at this temperature for 1 min, and then were cooled from the melt at 20 °C/min to 25 °C. Moreover, in this same equipment, the isothermal spherulitic growth rate was measured. The samples, placed between two glass slides, were heated to 185 °C and kept at this temperature for 1 min to erase thermal history. The samples were then cooled at 50 °C/min to a temperature at which the spherulites began to appear, and the growth of the spherulite was followed isothermally as a function of time by recording micrographs. This procedure was repeated for different temperatures. For each temperature, the radius of the spherulites was measured and reported graphically as a function of time. In this way, it was possible to calculate the growth rate of the spherulites, and the experimental values were fitted using the Lauritzen-Hoffman equation.

## 3 Results and Discussion

The five PHBs samples involved in this work were investigated by ^1^H NMR spectroscopy, thermal gravimetric analysis (TGA), differential scanning calorimetry (DSC), polarized light optical microscope (PLOM), and wide-angle X-ray scattering (WAXS).

### 3.1 NMR and TGA Results

Comparing the ^1^H NMR spectra of the bacterial and synthetic PHBs (Figure S1) showed no detectable differences in stereochemistry, as expected due to the high stereoregularity of these PHBs. Likewise, degradation profiles of these five PHBs revealed by TGA (Figure S2) were similar, showing no weight loss below 200 °C. To avoid problems of possible sample degradation, temperatures higher than 190 °C were never used in all the analyses carried out in this study.

### 3.2 Nonisothermal DSC and Thermal Stability Results

**Figure 1:**
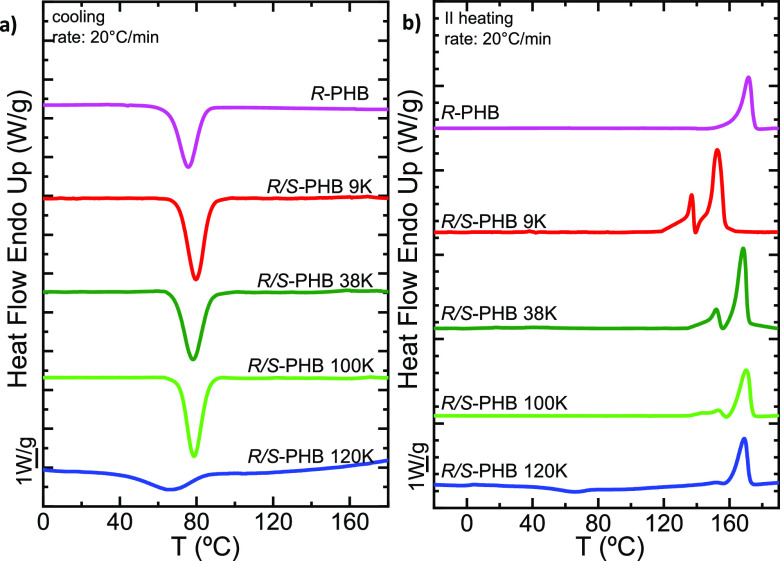
(a) DSC cooling scans at 20 °C/min and (b) subsequent DSC heating scans at 20 °C/min for bacterial *R* and synthetic *R*/*S*-PHBs.

The results of the nonisothermal characterization are shown in Figure 1. The samples belonging to *R*/*S*-PHBs (denoted as a function of their molecular weight as 9, 38, 100, and 120 K) showed no substantial differences during cooling compared to bacterial *R*-PHB 240 K, with the exception of *R*/*S*-PHB 120 K, which had a lower peak crystallization temperature (*T*_c_) and a smaller and broader crystallization exotherm. In fact, during the cooling scan in Figure 1a, the *R*/*S*-PHB 120 K sample did not crystallize to saturation, and during the second heating scan, cold crystallization was observed (see Figure 1b).

**Figure 2:**
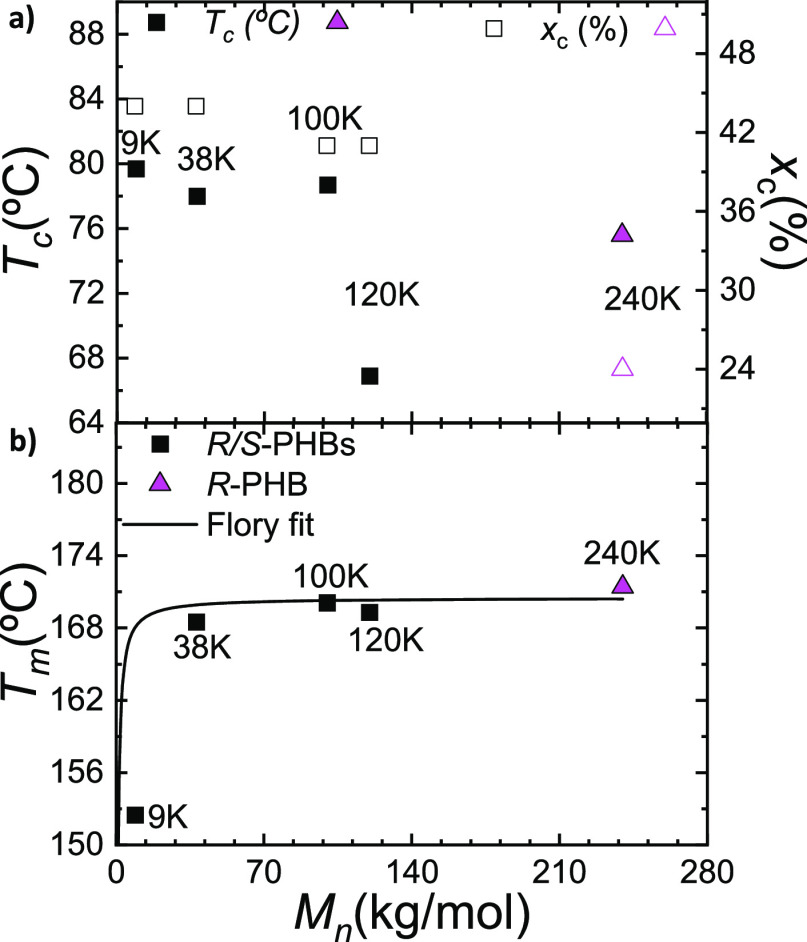
Crystallinity degree, crystallization (a), and melting (b) temperatures as a function of the molecular weight of the synthetic *R*/*S* (black squares) and bacterial *R* (pink triangle) PHBs; the solid black line in the bottom plot is a fit to Flory’s equation for the experimental *T*_m_ values.

Figure 2a represents the *T*_c_ and the *x*_c_ values extracted from the nonisothermal DSC scan and plotted as a function of molecular weight. The degree of crystallinity was calculated from the DSC cooling scan, as indicated in the SI.

The *T*_c_ values during cooling from the melt are influenced by the nonisothermal crystallization kinetics and by the nucleating influence of heterogeneities. As the same synthetic methods and reagents were employed, we assumed the nucleating influence of heterogeneities is constant, at least within the *R*/*S*-PHB synthetic samples. The trend is similar for both parameters in the case of the *R*/*S*-PHBs as both the degree of crystallinity and the *T*_c_ decreases as a function of molecular weight. Therefore, the lowest values of both the *T*_c_ and degree of crystallinity were found in the *R/S*-PHB with the highest molecular weight prepared (i.e., 120 kg/mol). The highest molecular weight *R*/*S*-PHB chains require more time to crystallize during cooling from the melt at 20 °C/min (as their diffusion may be limited due to a higher entanglement density and thus higher melt viscosity).

On the other hand, the bacterial *R*-PHB 240 K, even though it has a higher molecular weight than the *R*/*S*-PHB 120 K sample, showed relatively high *T*_c_ and *x*_c_ values (Figure 2a) and no significant cold crystallization during the second heating scan (Figure 1b). This strikingly different behavior is attributed to the sample’s enantiomeric purity, which has an all-*R* chain configuration that can crystallize faster under nonisothermal conditions than the *R*/*S*-PBH 120 K sample when cooling from the melt, despite its higher molecular weight. The 50% content of *S* chains in the *R*/*S*-PHB racemic mixture must be slowing down the nonisothermal crystallization from the melt.

Figure 2b shows a plot of the peak *T*_m_ values obtained during the second DSC heating scans shown in Figure 1b (the highest melting peaks were employed for the plot). As all the *R*/*S*-PHB samples exhibited a complex fusion behavior (Figure 1b) where a small, lower *T*_m_ peak can be seen, followed by a second, more pronounced peak at higher temperatures, we performed in situ WAXS (Figure S3) to demonstrate that this small peak is due to a reorganization process where thinner lamellae melt, recrystallize, and finally melt at higher temperatures (see also Figure 4 and its discussion below). As expected, the *T*_m_ value is a function of molecular weight that saturates beyond a critical molecular weight. The results were fitted with Flory’s equation,^35,36^ written as
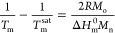
where *T*_m_^sat^ is the *T*_m_ when the saturation is reached (170.5 °C), *R* is the gas constant, Δ*H*_m_^0^ is the enthalpy of fusion at equilibrium,^37^ and *M*_o_ is the molecular weight of the repeating unit. See Table S1 for the values employed for the fitting of Flory’s equation.

### 3.3 Powder Diffraction and In Situ WAXS Real-Time Synchrotron Results

**Figure 3:**
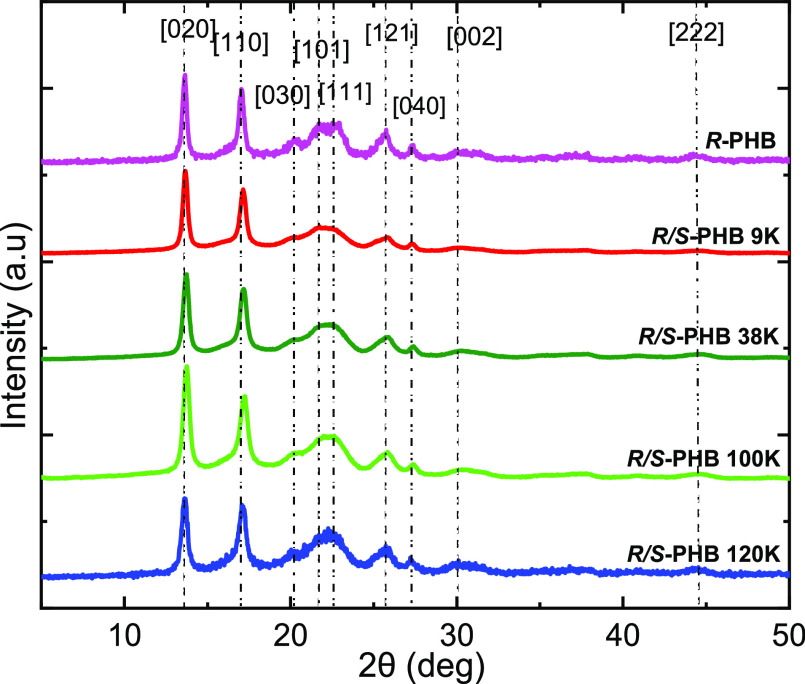
WAXS patterns taken at room temperature for the bacterial *R*-PHB and synthetic racemic *R*/*S*-PHBs as prepared. The planes that give origin to the reflections are indicated in the figure.

WAXS data at room temperature were collected for all the PHB as prepared samples, and the profiles obtained are shown in Figure 3, where the scattering intensity is reported as a function of 2θ, the diffraction angle. The diffraction profiles are very similar for all the samples, which indicates no differences in the crystalline structure between *R*-PHB and *R*/*S*-PHBs. Through Bragg’s law, it was possible to assign a Miller index to each peak, the relative data are reported in Tables S2–S6. These results indicate that, despite the different absolute stereoconfigurations, all the PHB samples employed here crystallize in an identical way to *R*-PHB,^38^ with an orthorhombic unit cell with the following dimensions: *a* = 5.76 Å, *b* = 13.30 Å, and *c* = 5.96 Å.

Overall, these results indicate that the racemic mixtures synthesized here with 50/50 *R*/*S* chains form a single phase in the crystalline state, which is identical to that of *R*-PHB.

As described above, the *R*/*S*-PHB samples exhibited two melting peaks and cold crystallization events (Figure 1). Therefore, we performed in situ WAXS studies by cooling the samples from the melt and then performed subsequent heating at the same rate used in the DSC studies at the Alba synchrotron for three representative samples: *R*-PHB and *R*/*S* PHBs (9 and 120 K) to ensure that the preceding small *T*_m_ peak, appeared before the considerably larger, higher *T*_m_ peak, was due to a reorganization process.

The WAXS diffraction spectra for cooling and heating scans are reported in Figures S4–S6, in which the intensity is reported as a function of the scattering vector *q*. The diffraction patterns are similar to those shown and described in Figure 3, and in them, the change in intensity of the peaks following crystallization and melting of the samples is clearly observed.

**Figure 4:**
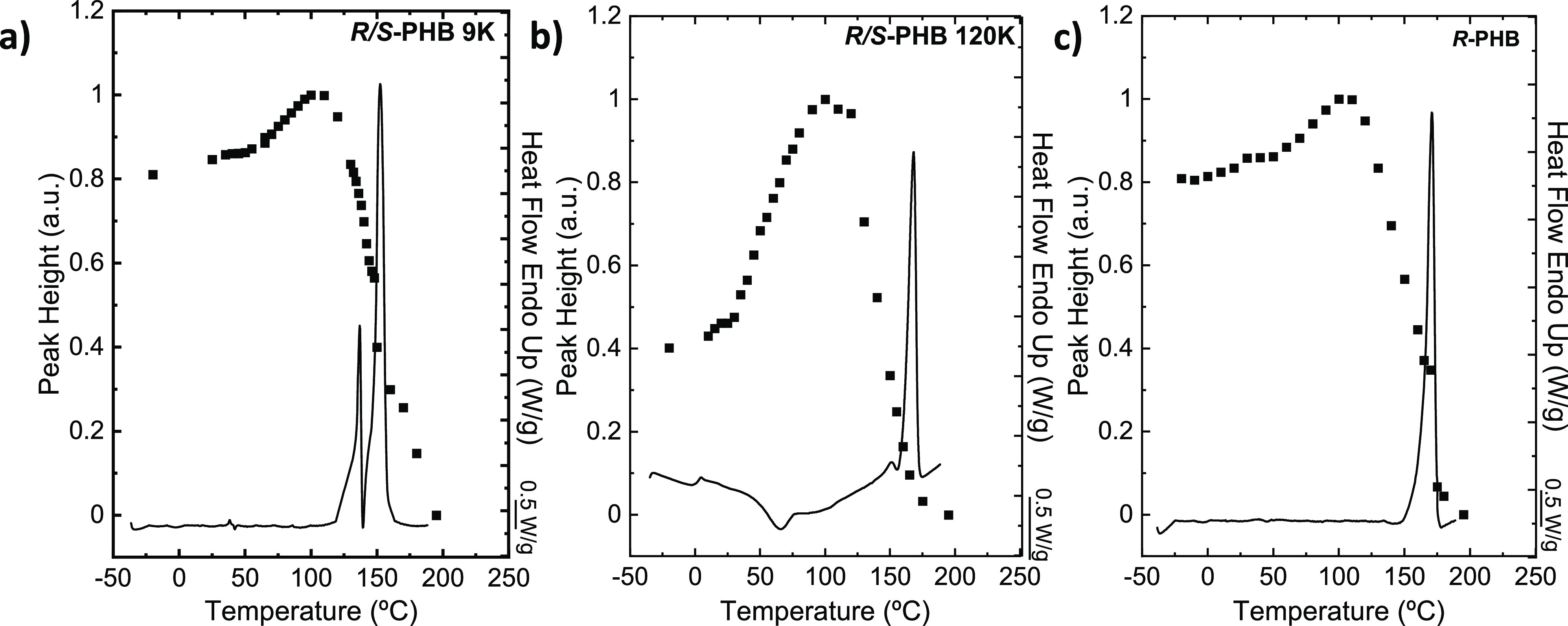
Normalized WAXS heating peak height intensities as a function of temperature for synthetic *R*/*S*-9K (a), *R*/*S*-120 K (b), and bacterial *R*-PHB (c), overlapped on the second heating curves from DSC.

To better analyze the nature of the double peak present in the DSC fusion scan, the intensity of the highest intensity diffraction peak was measured for the three samples mentioned earlier. The normalized intensity value acquired during the second heating scan is shown in Figure 4 as a function of temperature, for racemic *R*/*S*-PHB 9K (a), *R*/*S*-PHB 120 K (b), and bacterial *R*-PHB (c) samples.

Based on the DSC heating scan analysis, the phenomenon of cold crystallization can be observed clearly only for the racemic *R*/*S*-PHB 120 K sample. However, Figure 4 shows that the three samples examined, *R*/*S*-PHB 9K, *R*/*S*-PHB 120 K, and *R*-PHB all undergo cold-crystallization during heating as the relative intensity of the chosen reflection increase with temperature during heating. The *R*/*S*-PHB 9K only undergoes an approximate 20% relative increase in intensity during heating in the temperature range of approximately 50–100 °C, while the *R*/*S*-PHB 120 K sample (which shows a clear cold crystallization exotherm in this temperature range, as shown in Figure 4) exhibits a 60% relative increase in intensity. The peak intensity of the crystalline reflection chosen is proportional to the crystallinity degree.

For the *R*/*S*-PHB samples in Figure 4, the differences in cold crystallization behavior are due to their different molecular weights (9 vs 120 kg/mol). The lower molecular weight sample crystallizes much faster during the previous cooling process, hence it exhibits far less cold crystallization during heating.

In the case of bacterial PHB, despite having a higher molecular weight (240 kg/mol), it has an increase in relative intensity of only 20%, much lower than the *R*/*S*-PHB 120 K. We hypothesized that the reason behind this phenomenon is due to the enantiomeric purity of the bacterial PHB sample. Thus, as the pure *R* crystallizes faster during the previous cooling from the melt, no significant cold crystallization was observed during the second heating scan.

### 3.4 Morphology and Spherulite Growth

Besides DSC and WAXS experiments, the morphology and spherulite growth of different samples were analyzed by PLOM to get a better understanding of the effect of molecular weight and stereochemistry on the crystallization behavior.

A thermal stability study is presented in Figure S3, where the *T*_m_ and *T*_c_ values are plotted as a function of the number of measuring cycles in the DSC. A decrease of 1 to 1.5 °C is always observed when passing from the first to the third step; therefore, the sample degrades during the repeated scans. For this reason, we always employed a fresh sample for each measurement to try to minimize the effects of degradation in our measurements.

**Figure 5:**
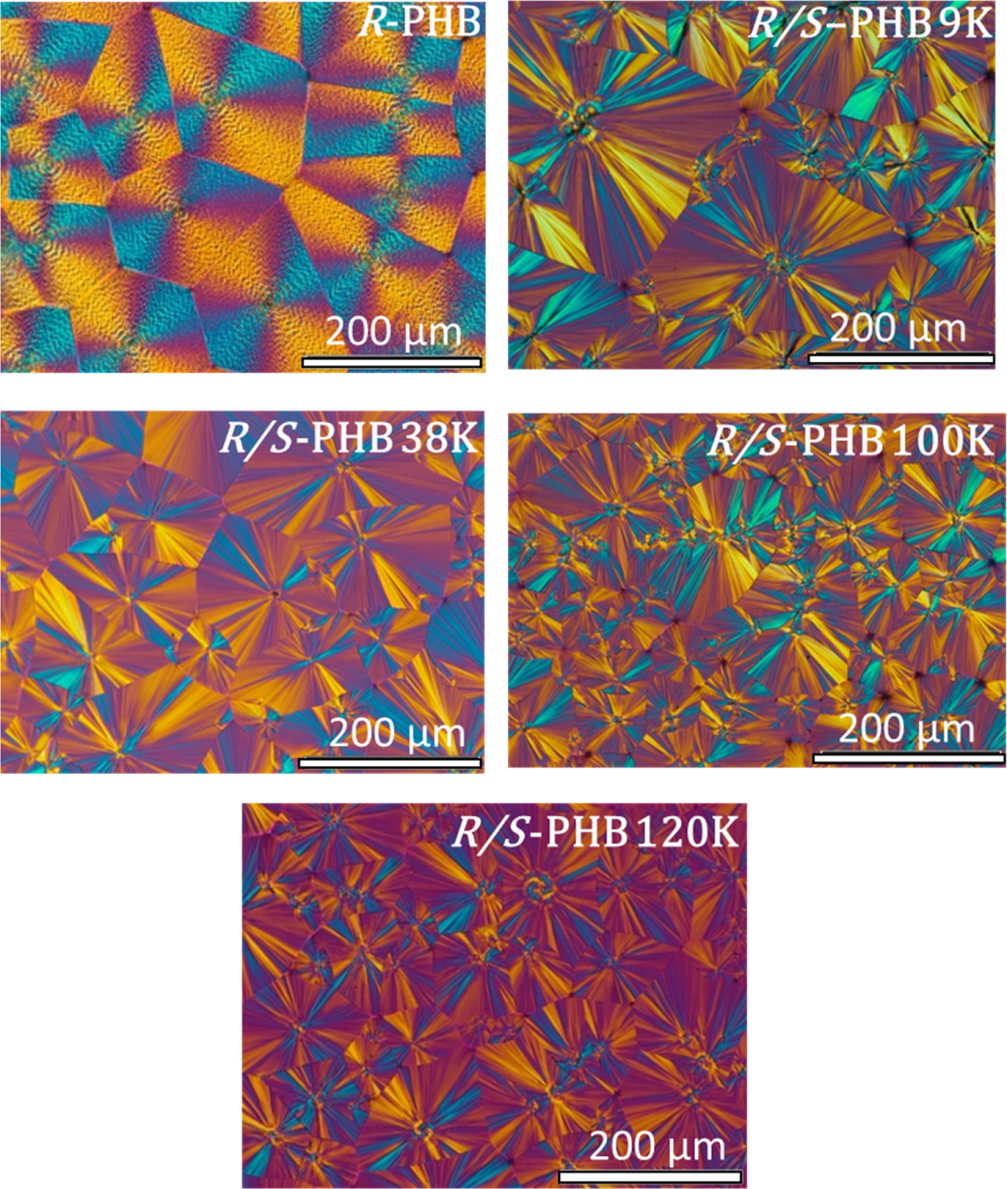
PLOM micrographs for bacterial and synthetic PHB samples. Micrographs were taken at 25 °C after melting for 1 min and cooling at 20 °C/min.

Figure 5 shows the PLOM micrographs of all PHB samples using the same magnification scale (200 μm). These micrographs were collected at 25 °C after nonisothermal crystallization from the melt at 20 °C/min. All samples crystallize to form spherulites, but with differences in the Maltese Cross extinction patterns. The bacterial *R*-PHB forms banded positive spherulites that agree with previous literature reports for the enantiomerically pure *R*-PHB.^37,39^ On the other hand, the racemic mixtures *R*/*S*-PHB samples form nonbanded spherulites, at least after nonisothermal crystallization at 20 °C/min. In addition, their spherulites reveal a mixed character where extinction patterns point toward a mixture of positive and negative spherulites, with a higher percentage of positive spherulites. Furthermore, spherulitic density (after nonisothermal crystallization) appears to moderately increase with molecular weight, especially from *R*/*S*- PHB 9 K to *R*/*S*-PHB 100 K, and then remains similar. These results imply a slight increase in nonisothermal nucleation density with molecular weight.

The isothermal growth of spherulites was determined by PLOM measurements. The samples were cooled from the melt (at 50 °C/min) to a chosen crystallization temperature in the range of 60 to 140 °C. Spherulitic growth rates for each sample, *G* (μm/s), were determined at different crystallization temperatures from the slope of radius versus time plots (which were always linear).

**Figure 6:**
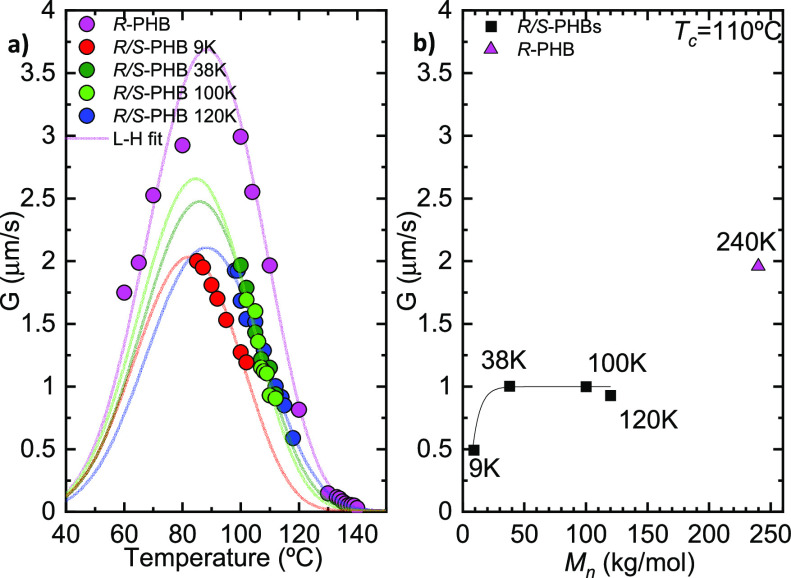
Spherulitic growth rate (*G*) as a function of (a) crystallization temperature and (b) molecular weight at *T*_c_ = 110 °C, for bacterial *R*-PHB, *R*/*S*-PHB 9, 38, 100, and 120 K. The solid lines in the left graph are fits to the Lauritzen and Hoffman equation. The solid line in the right plot is a line to guide the eye.

Figure 6a shows the spherulitic growth rates as a function of *T*_c_. The typical bell shape curve was obtained for *R*-PHB, which arises from the competition of two opposing trends.^40,41^ The growth rate increases as *T*_c_ decreases on the right-hand side of the plot as secondary nucleation increases with supercooling until a maximum is reached when the viscosity of the melt is so high that the growth of the crystals becomes dominated by the slow diffusion of the chains to the crystallizing front. Therefore, the growth rate decreases with supercooling and becomes zero at *T*_g_. On the other hand, in the case of the racemic synthetic *R*/*S*-PHB samples, it was not possible to measure growth rates in the left part of the curve because during the cooling process from the melt to *T*_c_ (at 50 °C/min), the samples crystallized until saturation in the observation area.

In Figure 6a, it is apparent that the growth rate value is higher in the enantiomerically pure bacterial *R*-PHB case. This aspect is peculiar because, given the higher molecular weight (240 kg/mol) of the sample compared to all synthetic others, one could have anticipated a lower growth rate. However, it appears that the enantiomeric purity is the dominating factor here and causing a faster growth rate despite the higher molecular weight of the sample. Therefore, it is apparent that the 50% content of *S*-PHB chains is slowing down the spherulitic growth rate of the *R*/*S* racemic mixture.

In the case of the racemic *R*/*S*-PHB synthetic samples, the growth rate is always lower than that of the enantiomerically pure *R*-PHB bacterial sample. Figure 6b shows how the growth rate depends on molar mass at the same *T*_c_ value (i.e., 110 °C). It can be seen that, within the family of racemic PHBs, the growth rate increases with the molecular mass and saturates at a value of approximately 1 μm/s (the slight decrease for the sample with 120 kg/mol in molecular weight is insignificant). However, the *R*-PHB sample has a growth rate of around 2 μm/s, so it grows twice as fast at this *T*_c_, presumably because of its enantiomerically pure *R* nature, even though its molecular weight is much higher (i.e., 240 kg/mol).

The solid lines in Figure 6a are fits to the Lauritzen and Hoffman theory. The detailed analysis of the fittings and the parameters obtained can be found in the Supporting Information (Table S7).

Small differences are noted for *K*_g_^*G*^ (constant proportional to the energy barrier for the spherulitic growth or secondary nucleation) and σ_e_ (fold surface energy) values in the family of *R*/*S* PHBs, in which these values increase slightly with molecular weight, probably due to the lower chain diffusion. The value of *q* (work that the macromolecule does to fold) also increases, and this could indicate that the chains require more energy to fold on the surface of the lamellae.^42^ For the sake of completeness, the values obtained by Barham et al. are also reported in the SI.^37^ The bacterial PHB used in the work of Barham et al. was a pure *R* sample but had a different molecular weight (133 kg/mol) than the *R*-PHB used in the present work. Although there are slight differences in the reported values, these are not significantly important as there are no differences in the growth rate *G* trend as a function of the *T*_c_ (see Figure S7).

**Figure 7:**
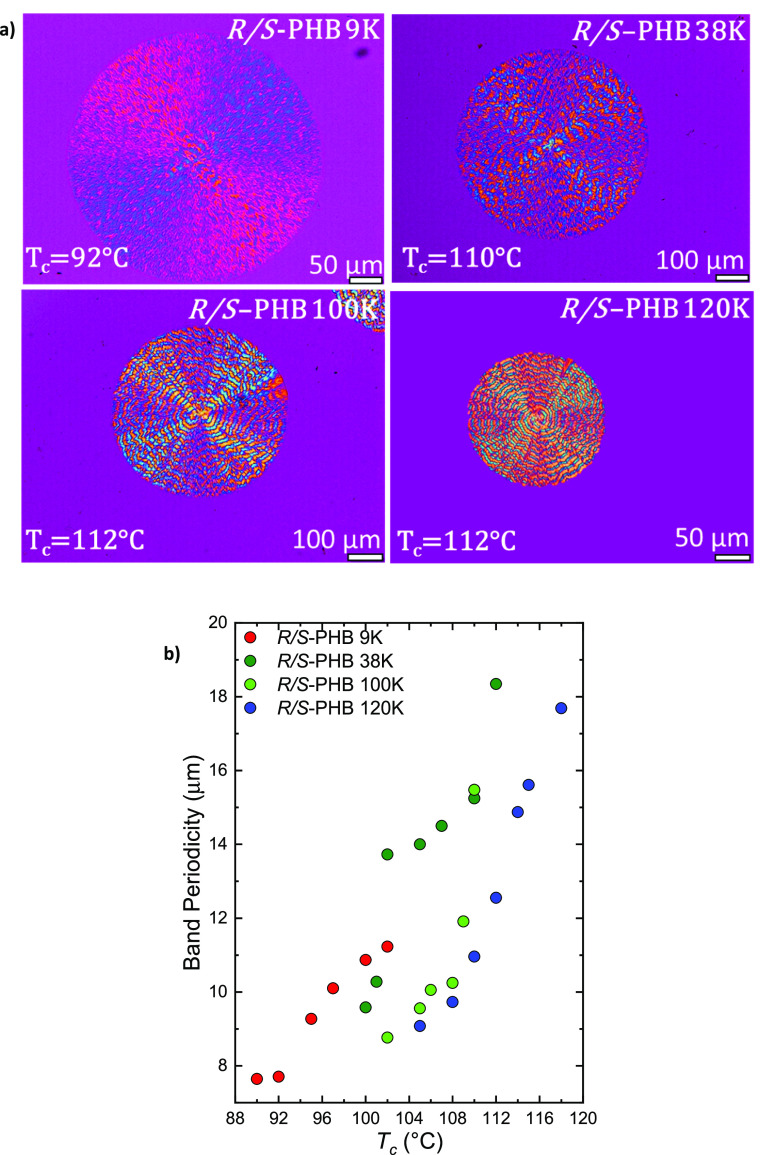
(a) PLOM micrographs taken at the indicated *T*_c_ and (b) band periodicity as a function of *T*_c_ for *R*-PHB, *R*/*S*-PHB 9, 38, 100, and 120 K.

From the isothermal spherulitic growth experiments, it was possible to observe banded spherulites for all the racemic synthetic PHB samples, even though in nonisothermal crystallization they did not form banded spherulites, presumably because of the relatively fast cooling rate (20 °C/min). It is well-known that banding periodicity in *R*-PHB is sensitive to *T*_c_, and its periodicity increases with *T*_c_. In Figure 7a, PLOM micrographs at different *T*_c_ are reported; they are collected at the indicated *T*_c_ values, and the bands in the spherulites are clearly visible in the case of the *R*/*S*-PHB samples. Through the ImageJ software, the band periodicity for different *T*_c_ values was calculated. Figure 7b shows how the band periodicity increases as *T*_c_ increases. This trend is similar to that reported for *R*-PHB in the literature.^37^

### 3.5 Study of the Overall Crystallization Kinetics by DSC

To study the overall crystallization kinetics (which includes both nucleation and growth contributions), isothermal crystallization experiments were performed by DSC. The obtained experimental data were analyzed with the Avrami theory and the Lauritzen and Hoffman theory.^43,44^

**Figure 8:**
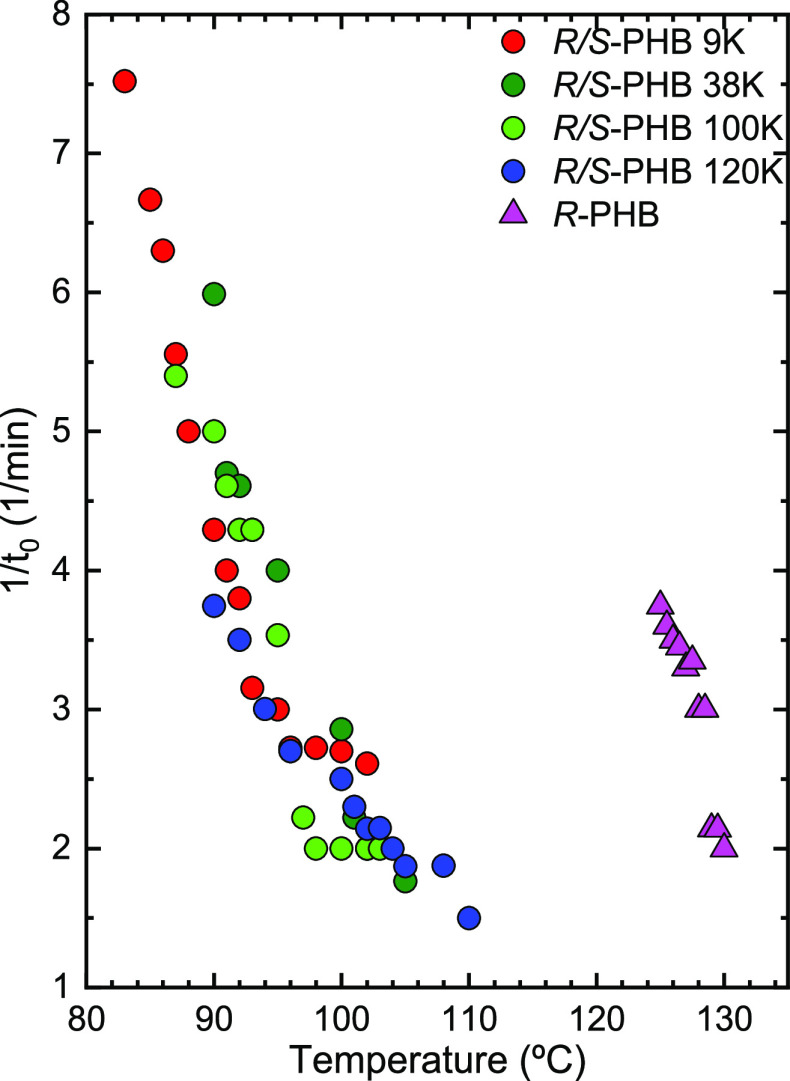
Inverse of induction time (1/*t*_0_) as a function of *T*_c_.

Figure 8 shows the inverse of the induction time (*t*_0_) as a function of *T*_c_ for the bacterial *R*-PHB and the synthetic racemic *R*/*S*-PHBs. The induction time is the primary nucleation time, which elapses before the DSC detects any crystallization process. Therefore, its inverse is proportional to the primary nucleation rate before crystallization starts. It can be seen that the bacterial *R*-PHB under isothermal conditions has a higher primary nucleation rate than the racemic *R*/*S*-PHBs if the trends of the plots are extrapolated as a function of *T*_c_, so that they can be compared at similar *T*_c_ values (for instance at 125 °C). Furthermore, the fact that *R*-PHB needs much lower supercooling to nucleate than all the *R*/*S*-PHB samples is also proof that enantiomerically pure *R*-PHB can nucleate faster than the former racemically mixed *R*/*S*-PHBs under isothermal conditions, even when its molecular weight is higher. This enhanced nucleation in the bacterial *R*-PHB is presumably due to the easier nucleation ability of *R*-PHB chains.

**Figure 9:**
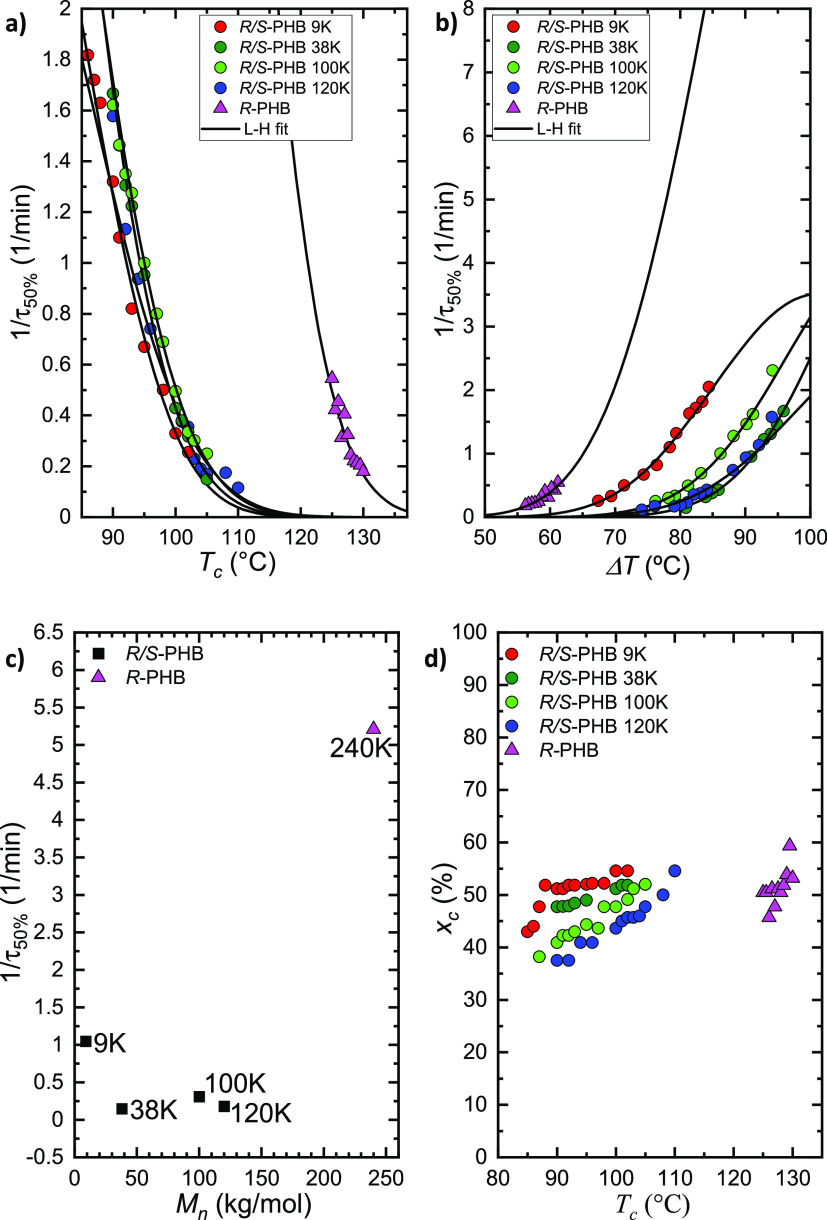
Inverse of half-crystallization time (1/τ_50%_) as a function of *T*_c_ (a), supercooling, Δ*T* (b), and *M*_n_ (c) at Δ*T* = 77 °C. Degree of crystallinity (*x*_c_) calculated during isothermal crystallization as a function of *T*_c_ (d). The solid lines in Figure 9 represent the fits to the Lauritzen and Hoffman theory.

The inverse of the half crystallization rate (1/τ_50%_) is plotted, in Figure 9, as a function of *T*_c_ (a), of the supercooling, Δ*T* (b), and molecular weight with the same Δ*T* = 77 °C (c). This value is the inverse of the time that, during an isothermal process, polymers need to achieve the 50% of their relative crystallinity. Moreover, this parameter represents the experimental overall crystallization rate, which considers both nucleation and growth contributions. The trend is similar to that seen in Figure 8 for the inverse of the induction time: bacterial *R*-PHB with a higher molecular weight (240 kg/mol), crystallizes faster and at lower supercooling than any of the *R*/*S*-PHB samples with lower molecular weights. Within the family of racemic *R*/*S*-PHB mixtures, slight differences are noted due to the molecular weight, even if the involved crystallization rates are lower, as shown in Figure 9a,b.

An important trend can be appreciated in Figure 9c, where 1/τ_50%_ is plotted as a function of molecular weight at a constant supercooling value of Δ*T* = 77 °C. It is observed that for the racemic synthetic *R*/*S*-PHB samples, the overall crystallization rate values are similar, within the limits of the experimental error compared to the bacterial *R*-PHB sample, which exhibits an overall crystallization rate (obtained by extrapolating the fit of the LH theory) that is remarkably five times faster than the lowest molecular weight *R*/*S*-PHB sample (i.e., 9 kg/mol). In the case of the growth rates determined above by PLOM, the increase in growth rates for the *R*-PHB with respect to *R*/*S*-PHBs went up to two times at specific *T*_c_ values (Figure 6b). This much higher overall isothermal crystallization rate (which measures both nucleation and growth) is an indication that not only is crystal growth faster, but also isothermal nucleation is faster at specific *T*_c_ values in enantiomerically pure *R*-PHB, something in line with the estimations of the nucleation rate in Figure 8. Although we could not rule out the potential effects of trace nuclearting impurities possibly present in the commercial bacterial sample (the synthetic samples were repeatedly purified until constant thermal property values were reached), the experimental evidence pointed to the conclusion that the stereochemistry is the decisive factor for the differences observed in the bacterial and synthetic PHB samples.

Figure 9d shows the degree of crystallinity (*x*_c_) obtained at the end of the isothermal crystallization process for all samples calculated as reported in the SI.

As can be seen from Figure 9d, the degree of crystallinity increases as *T*_c_ increases. Interestingly, in the *T*_c_ range of 90 to 105 °C, the crystallinity degree for the *R*/*S*-PHB samples increases as the molecular weight decreases. This trend is consistent with the slower diffusion ability of the higher molecular weight chains in the racemic mixtures. However, the enantiomerically pure *R*-PHB sample with a higher molecular weight (compare *R*/*S*-PHB 120 K with *R*-PHB 240 K) reaches similar degrees of crystallinity or slightly higher at lower supercooling dictated by the stereochemistry.

To fit the DSC overall crystallization rate experimental data, the Avrami theory^45−47^ was employed. The Avrami equation can be expressed as
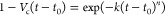
where *V*_c_ is the relative volumetric transformed fraction, *t* is the time of the experiment, *t*_0_ is the induction time already described above, *k* is the overall crystallization rate constant, and *n* is the Avrami index, related to the nucleation rate and the growth dimensionality of the crystals. In the case of bulk polymers, the values of *n* fluctuate between 2 and 4. If instantaneously nucleated axialites are obtained during primary crystallization, the corresponding *n* values would be 2 (which experimentally could fluctuate between 1.5 and 2.4). In the case of spherulites, the *n* values are close to 3 for instantaneous nucleation (i.e., 2.5–3.4) and 4 for sporadic nucleation (i.e., 3.5–4).

The Avrami theory usually fits the crystallization data in the primary crystallization regime, where the free growth of superstructural crystals (i.e., axialites or spherulites) is seen. After they start impinging one another, secondary crystallization sets in, and the Avrami equation cannot perfectly describe this complex process. In the case of *R*-PHB very few works are reported in the literature in which the overall crystallization kinetics have been studied.

Dubini et al.^48^ use the same conditions employed in this work, but the bacterial PHB used had a different molecular weight. An et al.^49^ and Gunaratne et al.^50^ studied the same *R*-PHB employed here but using different conditions, such as higher temperatures and times to erase the thermal history in the melt. Furthermore, none of the reported works performed isothermal crystallization experiments using fresh samples for each *T*_c_. This procedure could not prevent possible degradation of the sample during the experiments, as we demonstrated in Figure S3, in which a decrease in *T*_m_ and *T*_c_ was observed for successive cycles of crystallization and melting.

**Figure 10:**
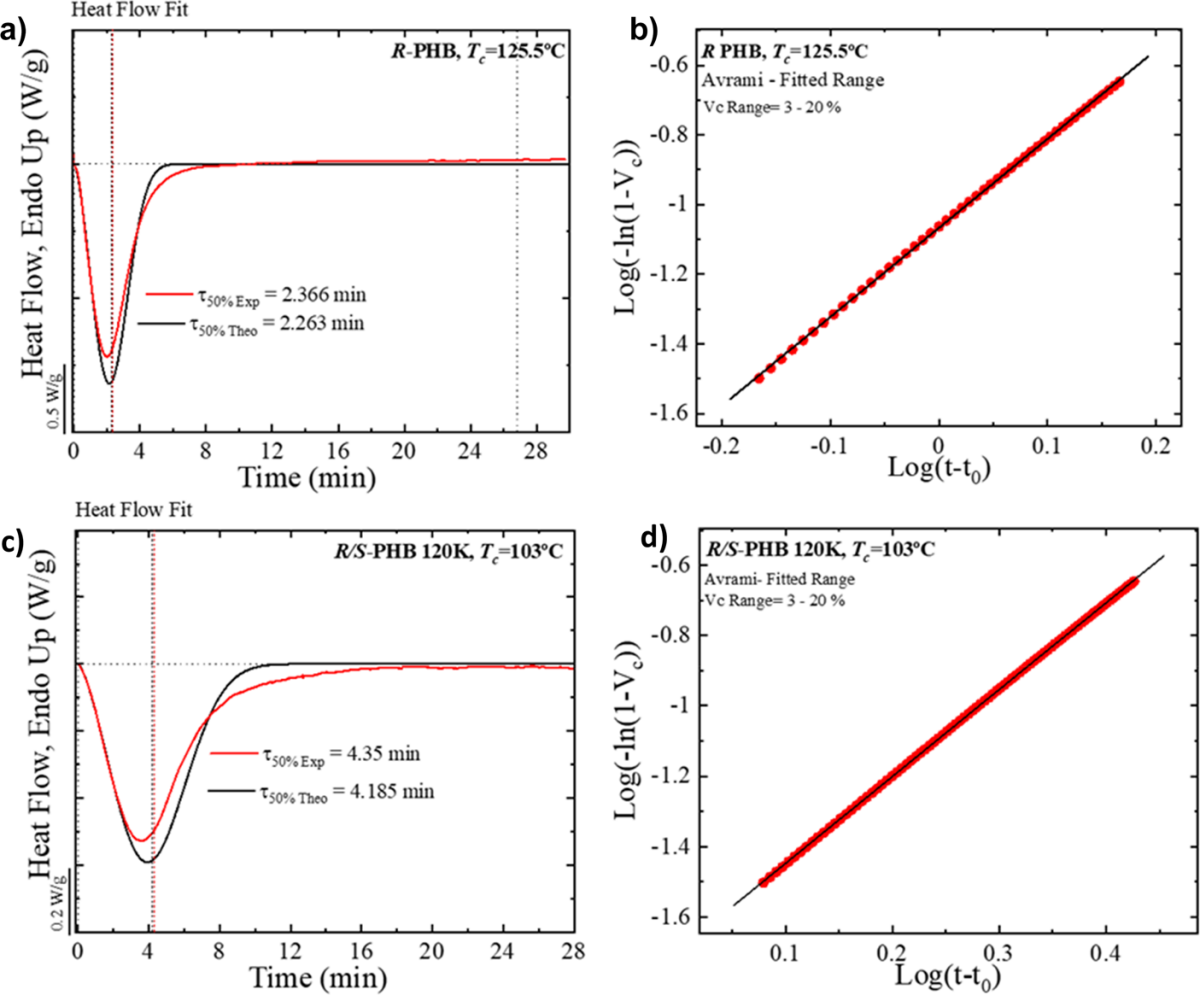
Avrami fit equation using the free Origin plug in developed by Pérez-Camargo et al.^51^ for bacterial *R*-PHB (a, b) and synthetic *R*/*S*-PHB 120 K (c, d) at the indicated *T*_c_.

Figure 10a,c presents two examples where the experimental DSC isotherms are plotted for *R*-PHB and for *R*/*S*-PHB 120 K together with the superposition of their respective Avrami fits. Figure 10c,d shows the typical Avrami plots in the conversion range (i.e., the relative crystallinity range) employed to perform the fit (3–20%) obtained by the free App developed by Pérez-Camargo et al.,^51^ which was used to perform the fittings. In the primary crystallization range, the fittings obtained are excellent, with correlation coefficients that are always larger than 0.999. Tables S9–S13 in the Supporting Information list the fitting parameters for all the samples employed here. Comparing the experimental values of τ_50%_ with those predicted by the Avrami theory, one can have an idea of whether the theory holds until 50% relative conversion to the semicrystalline state, and in this case, the agreement is very good.

**Figure 11:**
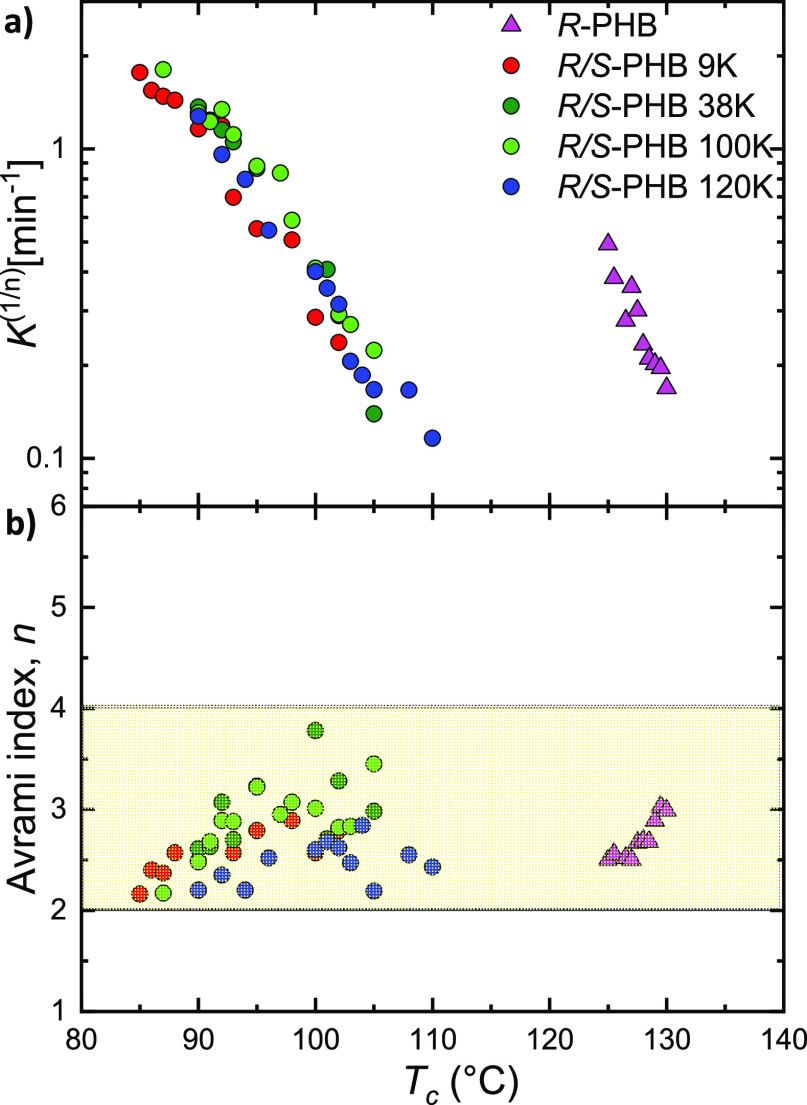
(a) Isothermal crystallization rate constant obtained by Avrami model (*k*^1/*n*^) and (b) Avrami index (*n*) as a function of *T*_c_ for bacterial *R*-PHB, *R*/*S*-PHB 9, 38, 100, and 120 K.

In Figure 11, the values of *k*^1/*n*^ (a) and *n* (b) are plotted as a function of *T*_c_, where *k*^1/*n*^ is directly proportional to the overall crystallization rate. The trend in Figure11a is very similar to the one obtained experimentally by measuring 1/τ_50%_ (Figure 10a), attesting for the good fit of the Avrami theory.

Regarding the Avrami index values shown in Figure 11b, it can be seen that they fluctuate from 2.1 to 4 for the *R*/*S*-PHB racemic synthetic samples and this indicates the presence of axialites for low crystallization temperatures and spherulites for high crystallization temperatures. For the bacterial *R*-PHB sample, *n* values between 2.5 and 4 were obtained, indicative of the presence of spherulites for the whole range of crystallization temperatures. These results are in good agreement with the observations performed by PLOM. Figure 11b shows that the Avrami index generally tends to increase with *T*_c_ for most samples. This trend can be explained when the morphology is fixed (i.e., for spherulites only or in the present case, when the Avrami index changes from 2.5 to 4) by the fact that the nucleation tends to change from instantaneous to sporadic as the temperature increases.^52^

### 3.6 Equilibrium Melting Point Determination

In the present work, during the isothermal crystallization process, the equilibrium melting temperatures (*T*_m_^0^) were calculated for the five PHB samples involved in the study. For this purpose, the Hoffman–Weeks extrapolation was used. In Figure S8, the extrapolations performed for all the PHB samples are shown.

Two endothermic peaks were recorded for the synthetic *R*/*S*-PHB racemic samples in the heating curves following the isothermal crystallization process. However, the temperatures chosen to extrapolate the *T*_m_^0^ were the ones derived from the first melting peaks, since they increase as *T*_c_ increases, and therefore, they represent the melting of the isothermally crystallized crystals. The second melting peak, obtained at high temperatures, is due to the melting of crystals reorganized during the heating scan and did not exhibit a significant variation of *T*_m_ with *T*_c_.

**Figure 12:**
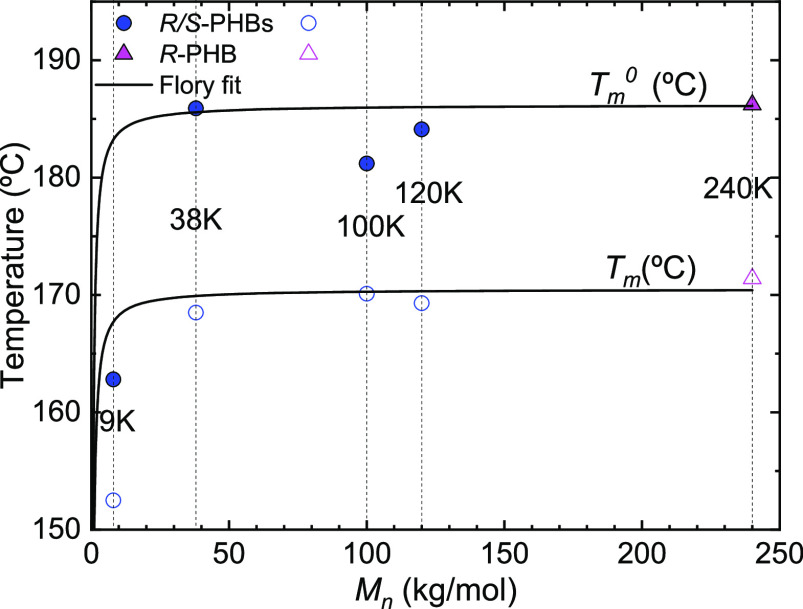
Melting temperature (*T*_m_) and equilibrium melting temperature (*T*_m_^0^) as a function of molecular weight for bacterial *R*-PHB, *R*/*S*-PHB 9, 38, 100, and 120 K; the solid black lines are Flory’s fit for the experimental points.

Figure 12 shows the *T*_m_^0^ values obtained as a function of the molecular weight of the samples. They are compared in the same plot with the apparent *T*_m_ measured by nonisothermal DSC experiments (reported in Figure 2b). The *T*_m_^0^ values were also fit with the Flory equation,^35,36^ and it is possible to note an increase of *T*_m_^0^ with the increase of molecular weight up to a saturation point. As expected, the *T*_m_^0^ values are larger than the apparent experimentally determined *T*_m_. The difference in this case is nearly 15 °C for most samples. No significant difference was found in the *T*_m_^0^ between the *R*-PHB sample and the *R*/*S* samples of higher molecular weights, that is, after a saturation value (185 °C) is obtained. The *T*_m_^0^ for the *R*-PHB of bacterial origin was previously measured in different works,^37,36,42,53,54^ and the values obtained are reported in Table S14, where a good agreement with the value obtained in the present work is noted.

## 4 Conclusions

In this study, we aimed to uncover the potential effect of the PHB stereochemistry on the crystallization behavior. To do so we performed an in-depth analysis of *R*-PHB with pure *R* stereoconfigurational structure and the analogous racemic mixture when blended with 50% of *S* chains. While we observed no effect of the stereochemistry in the equilibrium melting temperature, given the fact that the crystalline structure of both *R* and 50/50 *R*/*S*-PHB samples are also identical, we observed an important effect in the crystallization behavior. Indeed, a higher spherulitic growth rate and an even higher overall crystallization rate were found for the *R*-PHB sample, despite possessing a much higher molecular weight than all the 50/50 *R*/*S*-PHB racemic mixture samples employed here. Such enhancement in nucleation and growth can only be explained by the differences in enantiomeric character between the samples. The mixture of chains with both *R* and *S* chain configurations can slow down the primary nucleation and the growth of the chain ensemble during crystallization, relative to *R*-PHB chains with only *R* chain configuration. An implication of these results is that the use of the synthetic, racemic PHB could spread the use of PHB-based materials because its employment in the preparation of copolymers and blends with other polyesters could lead to new materials with a slower crystallization kinetics and a wider processability window.

## Supporting Information Available

Figures S1 and S2: ^1^H NMR spectra and TGA results; Table S1: Thermal parameters obtained from unstable crystallization and brief descriptions of its calculations; Figure S3: Trend of crystallization and melting temperatures as a function of the number of thermal cycles; Tables S2–S6 and Figures S4–S6: Structural parameters and relative WAXS diffraction profiles; Tables S7 and S8: Lauritzen-Hoffman equation’s description and isothermal kinetics data parameters obtained by PLOM and DSC; Figure S7: Comparison of the experimental growth rate of spherulites with the values reported in the literature; Tables S9–S13: Avrami fitting parameters; Figure S8: Hoffman–Weeks plots; Table S14: Equilibrium melting temperature values reported in the literature (PDF)
