# Supplementary material for: Effect of Chain Stereoconfiguration on Poly(3-hydroxybutyrate) Crystallization Kinetics
Source: Biomacromolecules. 2022 Aug 5;23(9):3847–59. doi: 10.1021/acs.biomac.2c00682 (PMC9472230; doi:10.1021/acs.biomac.2c00682)
Supplement: Supplementary file 1 — bm2c00682_si_001.pdf [file bm2c00682_si_001.pdf]

Supporting Information

Effect of chain stereoregularity on poly(hydroxybutyrate) crystallization kinetics

Maria Rosaria Caputo [a], Xiaoyan Tang[b], Andrea H. Westlie [b] , Haritz Sardon* [a], Eugene Y. X. Chen [b], Alejandro J. Müller* [a, c]

[a] POLYMAT and Department of Polymers and Advanced Materials: Physics, Chemistry and Technology, Faculty of Chemistry, University of the Basque Country UPV/EHU, Paseo Manuel de Lardizabal 3, 20018 Donostia-San Sebastián, Spain

[b]Department of Chemistry, Colorado State University, Fort Collins, Colorado, 80523-1872, United States

[c] IKERBASQUE, Basque Foundation for Science, Plaza Euskadi 5, 48009 Bilbao, Spain

*Corresponding authors: haritz.sardon@ehu.eus; eugene.chen@colostate.edu; alejandrojesus.muller@ehu.es

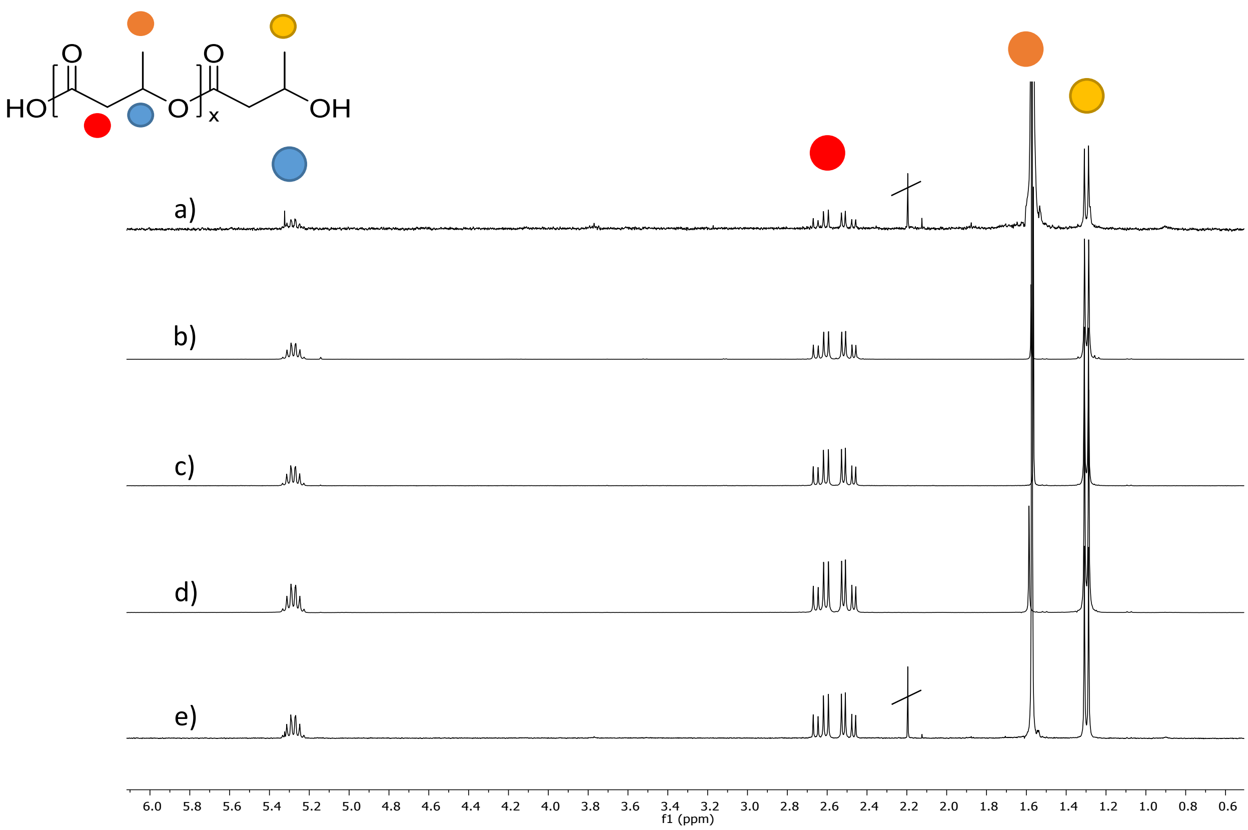

Figure S1. ^{1}H NMR spectroscopy in $CDCl_3$ of Bacterial *R* (a) and Synthetic *R/S*-PHBs, 8K (b) 38K (c), 100K (d), 120K (e).

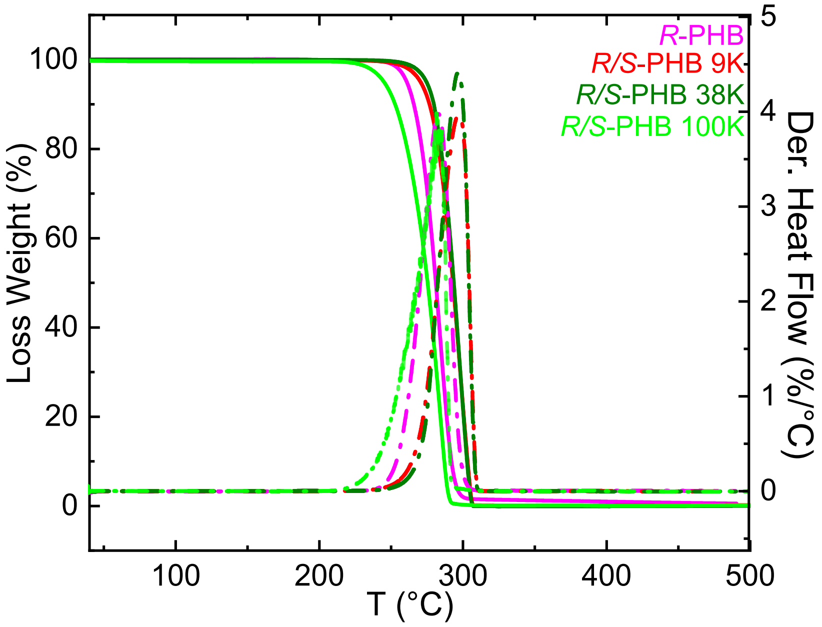

Figure S2. Loss Weight (%) and Derivate of Heat Flow (%/°C) as function of the temperature (°C) for Bacterial *R* and Synthetic *R/S* PHBs.

Table S1. Thermal DSC cooling and heating properties of Bacterial *R* PHB and Synthetic *R/S* PHB 8K, 38K, 100K and 120K.

***R*-PHB**	***R/S*-PHB 9K**	***R/S*-PHB 38K**	***R/S*-PHB 100K**	***R/S*-PHB 120K**
T_c=75.6°C<br>ΔH_c=65 J/g	T_c=79.7°C<br>ΔH_c=64 J/g	T_c=78.0°C<br>ΔH_c=60 J/g	T_c=78.7°C<br>ΔH_c=60 J/g	T_c=66.9°C<br>ΔH_c=35 J/g<br>T_{cc}=65.2°C<br>ΔH_{cc}=-11 J/g
T_m=171.4°C<br>ΔH_m=67 J/g	T_{mI}=136.8°C,<br>T_{mII}=152.5°C<br>ΔH_{mI}=19 J/g<br>ΔH_{mII}=100 J/g	T_{mI}=151.8°C,<br>T_{mII}=168.5°C<br>ΔH_{mI}=14 J/g<br>ΔH_{mII}=87 J/g	T_{mI}=153.1.°C,<br>T_{mII}=170.1°C<br>ΔH_{mI}=5 J/g<br>ΔH_{mII}=51 J/g	T_m=169.3°C<br>ΔH_m=60 J/g
T_g=4°C				T_g=1.4°C
x_c=44%	x_c=44%	x_c=41%	x_c=41%	x_c=24%

The degree of crystallinity was calculated from the DSC cooling scan as follows:

$$x_c = \frac{\Delta H_c}{\Delta H_m^0} \times 100$$

where ΔH_c is the crystallization enthalpy and ΔH_m^0 is the enthalpy of fusion at equilibrium (146 J/g,[1]).

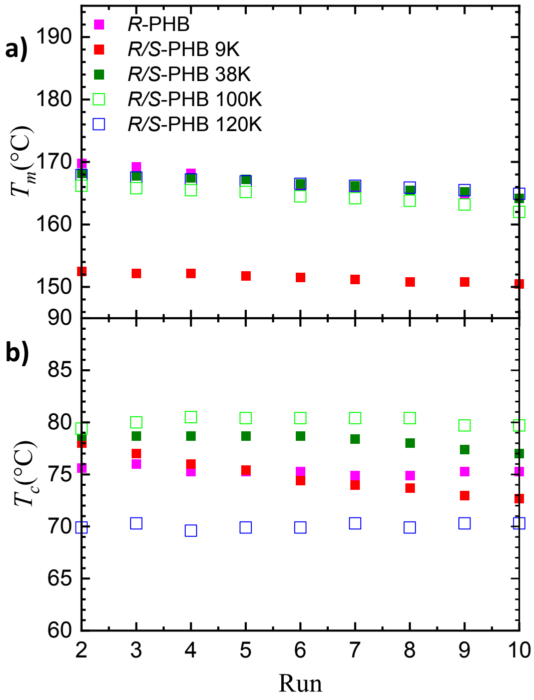

Figure S3. a) Melting point (T_m) and and b) crystallization point (T_c) versus the number of run (stability test) for Bacterial *R* and Synthetic *R/S* PHB.

Table S2.Diffraction Angle (2θ), interplanar experimental (d exp) and calculated (d calc) distance and Miller index for Bacterial *R* PHB.

**2θ exp (deg)**	**d exp (Å)**	**d calc (Å)**	**Miller index**
13.64	6.492	6.600	020
17.03	5.206	5.760	110
20.24	4.387	4.400	030
21.73	4.089	4.141	101
22.89	3.885	3.951	111
25.75	3.459	3.508	121
27.28	3.269	3.300	040
29.90	2.988	2.980	002
44.03	2.056	1.975	222

Table S3.Diffraction Angle (2θ), interplanar experimental (d exp) and calculated (d calc) distance and Miller index for Synthetic *R/S*-PHB 9K.

**2θ exp (deg)**	**d exp (Å)**	**d calc (Å)**	**Miller index**
13.68	6.472	6.600	020
17.09	5.188	5.760	110
20.03	4.433	4.400	030
21.80	4.076	4.141	101
22.77	3.905	3.951	111
25.72	3.463	3.508	121
30.07	3.255	2.980	002
44.70	2.027	1.975	222

Table S4.Diffraction Angle (2θ), interplanar experimental (d exp) and calculated (d calc) distance and Miller index for Synthetic *R/S*-PHB 38K.

**2θ exp (deg)**	**d exp (Å)**	**d calc (Å)**	**Miller index**
13.73	6.449	6.600	020
17.17	5.164	5.760	110
19.96	4.448	4.400	030
21.74	4.087	4.141	101
22.45	3.960	3.951	111
25.92	3.437	3.508	121
29.88	3.255	2.980	002
44.70	2.027	1.975	222

Table S5.Diffraction Angle (2θ), interplanar experimental (d exp) and calculated (d calc) distance and Miller index for Synthetic *R/S*-PHB 100K.

**2θ exp (deg)**	**d exp (Å)**	**d calc (Å)**	**Miller index**
13.73	6.449	6.600	020
17.17	5.164	5.760	110
20.11	4.415	4.400	030
21.64	4.106	4.141	101
22.40	3.969	3.951	111
25.66	3.471	3.508	121
27.40	3.278	3.300	040
29.99	2.979	2.980	002
44.60	2.0315	1.975	222

Table S6.Diffraction Angle (2θ), interplanar experimental (d exp) and calculated (d calc) distance and Miller index for Synthetic *R/S*-PHB 120K.

2θ exp (deg)	d exp (Å)	d calc (Å)	Miller index
13.62	6.501	6.600	020
17.06	5.197	5.760	110
20.02	4.435	4.400	030
22.05	4.031	4.141	101
25.72	3.463	3.508	121
27.40	3.278	3.300	040
30.11	2.979	3.300	002
44.16	2.051	1.975	222

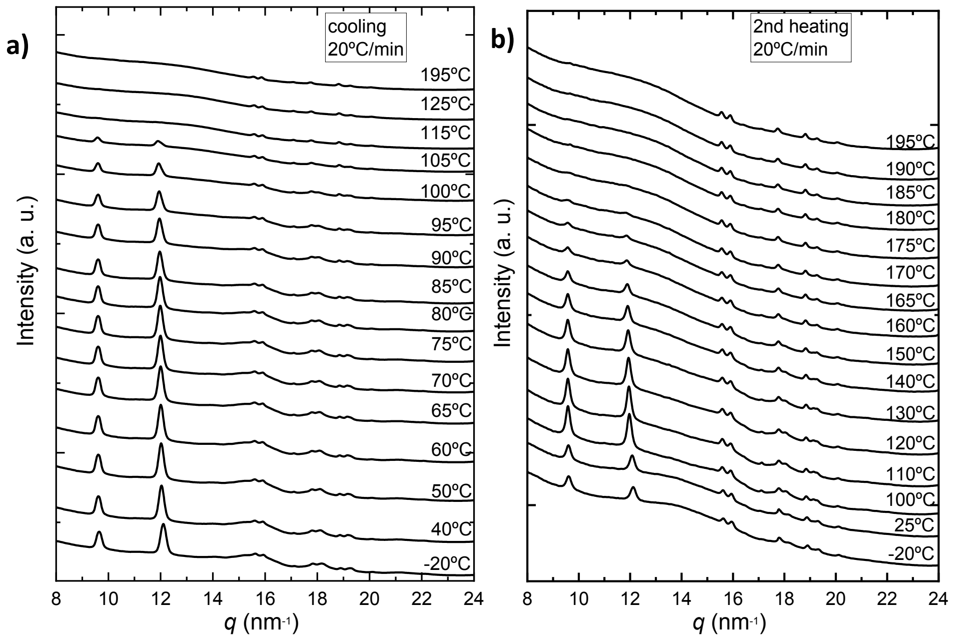

Figure S4 WAXS diffractograms, at different temperatures, acquired during the cooling (a) and heating scan (b) for Bacterial *R*-PHB.

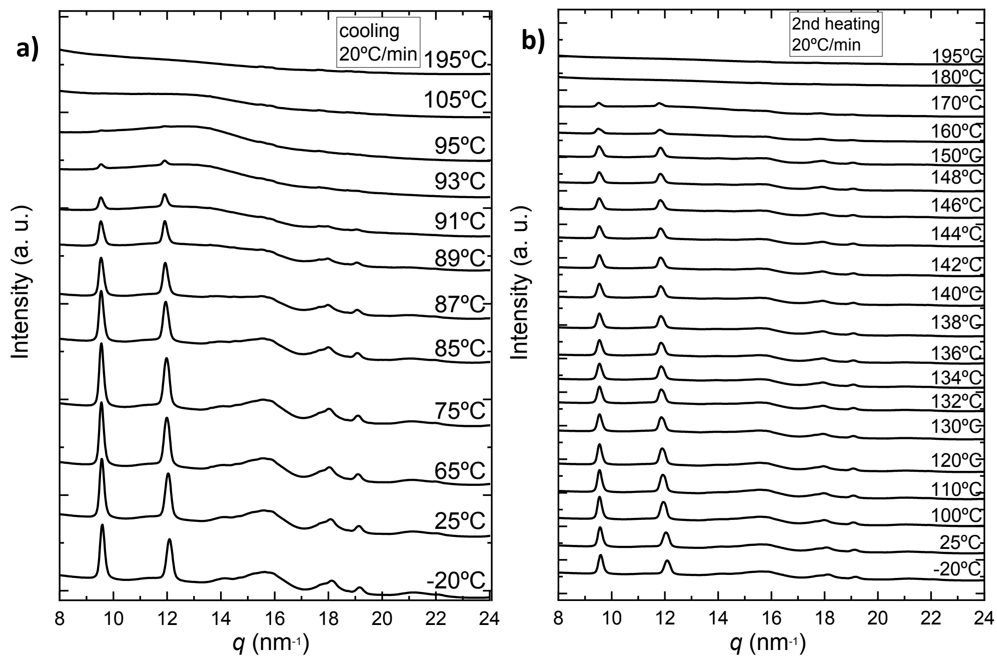

Figure S5 WAXS diffractograms, at different temperatures, acquired during the cooling (a) and heating scan (b) for *R/S*-PHB 9K.

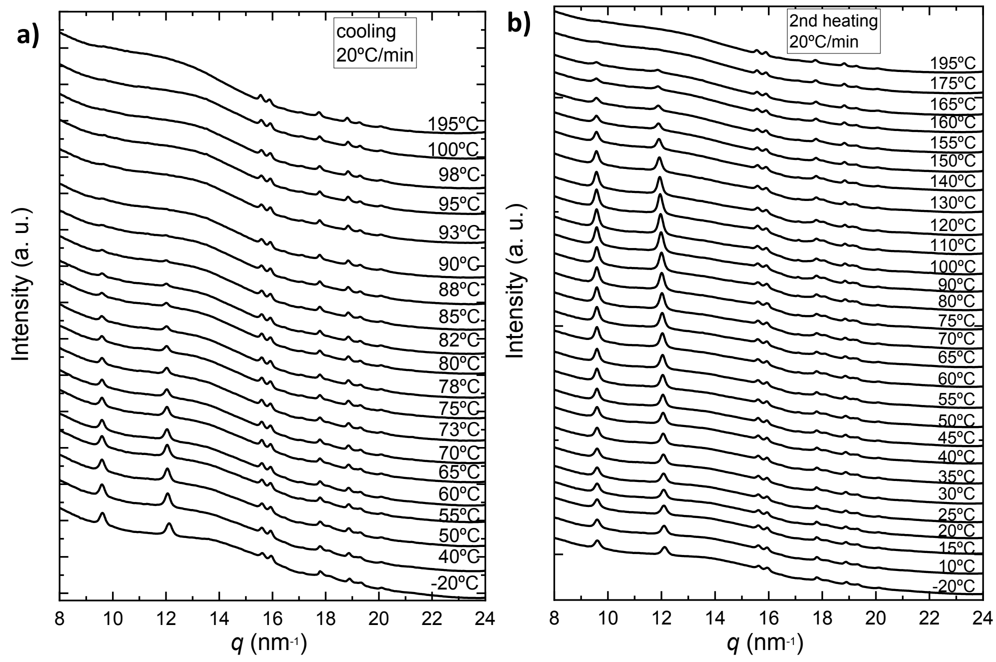

Figure S6 WAXS diffractograms, at different temperatures, acquired during the cooling (a) and heating scan (b) for *R/S*-PHB 120K.

The Lauritzen-Hoffman equation is given by[2]:

$$G = G_o\, exp\left[\frac{-U^*}{R(T_c - T_0)}\right]\left[\frac{-K_g^G}{fT(T_m^0 - T_c)}\right]$$

where: G_o is a constant that includes all terms that do not depend on temperature, U^* is the transport activation energy for the polymer chains diffusion (in this work, a value of 1500 cal/mol was employed), R is the universal gas constant, T_c is the crystallization temperature, T_0 is the temperature at which the movement of the chains is frozen, and it is 30ºC degrees lower than the T_g, T_m^0 is the equilibrium melting temperature, f is a temperature correction factor, given by the expression $2T_c/(T_m^0 + T_c)$. K_g^G is a constant proportional to the energy barrier for the spherulitic growth or secondary nucleation:

$$K_g^G = \frac{j\, b_0\, \sigma\sigma_e T_m^0}{k\, \Delta H_m^0}$$

j assumes the value of 2 for the Regime II crystallization, a regime where the secondary nucleation and the spread of the nucleus on the growth front are equivalent [3], b_0 is the chain's width, σ the lateral surface free energy, σ_e is the fold surface energy, k is the Boltzman constant and, finally, ΔH_m^0 is the equilibrium latent heat of fusion. When $\ln G + \frac{-U}{R\,(T_c - T_0)}$ is plotted versus $\frac{1}{T_c(\Delta T)f}$, it is possible to obtain a straight line in which K_g^G is the slope and G_o the intercept. From the K_g^G value, it is possible to calculate the $\sigma\sigma_e$ value, and, using the expression $\sigma = 0.1\Delta H_m^0\sqrt{a_0 b_0}$, where $a_0 b_0$ is the chain cross sectional area, it is possible to obtain the values of σ and σ_e. Moreover, it is also possible to calculate the work that the macromolecule does to fold as $q = 2a_0 b_0 \sigma_e$ [4].

Table S7. Isothermal kinetics data parameters described in the Paragraph 3.4 derived from experimental results obtained by PLOM. The last row shows the data present in the literature [1].

Sample	G^0(cm/s)	K^G_g (K^2)	σ (erg/cm²)	σ_e (erg/cm²)	q (erg)	R^2
R-PHB	70	3.0×10^5	8.39	256.4	9.46×10^{-13}	0.997
R/S-PHB 9K	27.6	2.47×10^5	8.39	218.7	8.07×10^{-13}	0.999
R/S-PHB 38K	136	3.33×10^5	8.39	284.2	1.05×10^{-12}	0.986
R/S-PHB 100K	154	3.42×10^5	8.39	285.4	1.15×10^{-12}	0.996
R/S-PHB 120K	230	3.8×10^5	8.43	320.9	1.18×10^{-12}	0.995
Barham *et al*. PHB[1]	121	4.9×10^5	8.35	359.7	1.32×10^{-12}	0.989

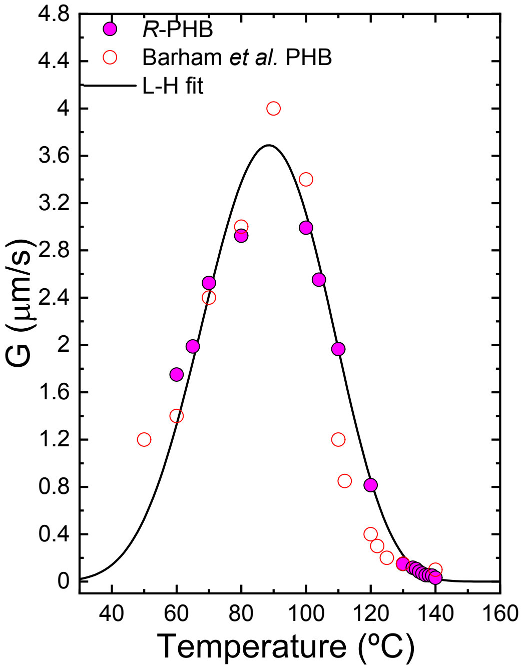

Figure S7 Spherulitic growth rate (*G*) as a function: of crystallization temperature for R-PHB studied in this work (filled pink circles) and for R-PHB reported in literature [1] (red empty circles).

The Lauritzen and Hoffman theory [2] can also be used to fit the overall crystallization data described in the Paragraph 3.5 and obtained from DSC. The equation, in this case, should be adapted as follows:

$$\frac{1}{\tau_{50\%}} = \frac{1}{\tau_{50\%}} exp\left[\frac{U}{R(T_c - T_0)}\right]\left[\frac{-K_g^{\tau}}{fT(T_m^0 - T_c)}\right]$$

The terms of this equation have been described in the Paragraph 3.4, except K_g^{τ} that; in this case, the constant is proportional to the energy barrier for both primary nucleation and spherulitic growth, unlike K_g^G that refers just to the energy barrier for the secondary nucleation or growth.

Table S8. Overall isothermal kinetics data parameters described in the Paragraph 3.5 derived from experimental results obtained by DSC

**Sample**	***1/τ₀*(1/s)**	***Kτg (K²)***	**σ (erg/cm²)**	**σe (erg/cm²)**	**q (erg)**	**R²**
R-PHB	$9.92x10^{7}$	$4.11x10^{5}$	8.39	350.9	$1.30x10^{-12}$	0.992
R/S-PHB 9K	$1.90x10^{8}$	$4.42x10^{5}$	8.39	353.5	$1.30x10^{-12}$	0.997
R/S-PHB 38K	$7.22x10^{12}$	$6.20x10^{5}$	8.39	699.9	$2.58x10^{-12}$	0.990
R/S-PHB 100K	$1.12x10^{12}$	$6.61x10^{5}$	8.39	570.24	$2.10x10^{-12}$	0.992
R/S-PHB 120K	$3.31x10^{10}$	$6.52x10^{5}$	8.39	558.69	$2.06x10^{-12}$	0.990

Table S9 Avrami fitting parameters obtained from by the free App [5] for *R*-PHB.

T_c(°C)	t_0(s)	$\tau_{50\%}$ Exp (min)	$\tau_{50\%\ Theo}$ (min)	n	$K^{\frac{1}{n}}$(min^{-1})	K (min^{-n})	R^2
125	0.267	1.833	1.754	2.5	0.492	1.70x10^{-1}	0.999
125.5	0.263	2.366	2.263	2.56	0.383	8.58x10^{-2}	0.999
126	0.285	2.199	2.034	2.54	0.301	1.14E-02	0.999
126.5	0.289	3.200	3.102	2.52	0.279	1.36x10^{-1}	0.999
127	0.303	2.467	2.327	2.50	0.358	7.70x10^{-2}	0.999
127.5	0.298	3.084	2.898	2.67	0.300	4.04x10^{-2}	0.999
128	0.333	4.083	3.729	2.69	0.233	2.00x10^{-2}	0.999
128.5	0.333	4.516	4.149	2.68	0.210	1.53x10^{-2}	0.999
129	0.467	4.682	4.525	2.89	0.202	2.47x10^{-2}	0.999
129.5	0.467	4.883	4.683	3.03	0.196	7.17x10^{-3}	0.999
130	0.501	5.566	5.236	2.99	0.168	4.91x10^{-3}	0.999

Table S10 Avrami fitting parameters obtained from by the free App [5] for *R/S*-PHB 9K.

T_c (°C)	t_0(s)	$\tau_{50\%}$ Exp (min)	$\tau_{50\%}$ Theo (min)	n	$K^{\frac{1}{n}}$(min^{-1})	K (min^{-n})	R^2
83	0.133	0.465	0.450	2.20	1.780	8.13	0.999
85	0.150	0.487	0.477	2.16	1.766	3.42	0.999
86	0.158	0.550	0.555	2.4	1.544	2.84	0.999
87	0.180	0.581	0.579	2.37	1.479	2.53	0.999
88	0.20	0.613	0.604	2.57	1.437	2.54	0.999
90	0.233	0.757	0.743	2.49	1.160	1.45	0.999
91	0.250	0.909	0.934	2.52	1.162	8.81x10^{-1}	0.999
92	0.263	0.987	0.972	2.89	1.189	1.65	0.999
93	0.317	1.219	1.215	2.57	0.698	3.97x10^{-1}	0.999

95	0.333	1.492	1.585	2.79	0.552	1.91x10^{-1}	0.999
96	0.367	1.666	1.650	2.82	0.525	2.21x10^{-1}	0.999
98	0.367	2.021	1.985	2.89	0.508	7.70x10^{-2}	0.999
100	0.370	3.032	3.103	2.57	0.285	2.28x10^{-2}	0.999
102	0.383	3.916	3.788	2.79	0.237	7.28x10^{-3}	0.999

Table S11 Avrami fitting parameters obtained from by the free App [5] for *R/S*-PHB 38K.

T_c (°C)	t_0(s)	$\tau_{50\%}$ Exp (min)	$\tau_{50\%}$ Theo (min)	n	$K^{\frac{1}{n}}(min^{-1})$	$K\ (min^{-n})$	R^2
90	0.167	0.599	0.602	2.61	1.366	2.26	0.999
91	0.212	0.683	0.702	2.63	1.239	1.76	0.999
92	0.217	0.766	0.771	3.07	1.151	1.54	0.999
93	0.233	0.817	0.830	2.7	1.053	1.15	0.999
95	0.250	1.050	1.032	3.23	0.864	6.25x10^{-1}	0.999
100	0.350	2.330	2.255	3.78	0.40195	3.19x10^{-2}	0.999
101	0.450	2.631	2.526	2.71	0.40769	8.79x10^{-2}	0.999
102	0.501	3.150	3.086	3.28	0.28925	1.71x10^{-2}	0.999
105	0.567	6.749	6.330	2.98	0.13927	2.81x10^{-3}	0.999

Table S12 Avrami fitting parameters obtained from by the free App [5] for *R/S*-PHB 100K.

T_c (ºC)	t_0(s)	$\tau_{50\%}$ Exp (min)	$\tau_{50\%}$ Theo (min)	n	$K^{\frac{1}{n}}(min^{-1})$	K (min^{-n})	R^2
87	0.18519	0.432	0.468	2.17	1.804	3.60	0.999
90	0.200	0.616	0.659	2.48	1.309	1.95	0.999
91	0.217	0.682	0.691	2.68	1.224	1.72	0.999
92	0.233	0.740	0.760	2.89	1.342	2.37	0.999
93	0.233	0.784	0.792	2.88	1.112	1.36	0.999
95	0.283	1.000	1.015	3.22	0.879	5.88×10^{-1}	0.999
97	0.450	1.250	1.105	2.95	0.835	1.96×10^{-1}	0.999
98	0.500	1.449	1.508	3.07	0.588	6.93×10^{-2}	0.999
100	0.500	2.016	2.151	3.01	0.411	3.11×10^{-2}	0.999
102	0.500	2.984	3.007	2.82	0.292	2.48×10^{-2}	0.999
103	0.500	3.316	3.242	2.83	0.270	5.68×10^{-3}	0.999

Table S13 Avrami fitting parameters obtained from by the free App [5] for *R/S*-PHB 120K.

T_c (ºC)	t_0(s)	$\tau_{50\%}$ Exp (min)	$\tau_{50\%}$ Theo (min)	n	$K^{\frac{1}{n}}(min^{-1})$	K (min^{-n})	R^2
90	0.267	0.633	0.615	2.20	1.276	1.74	0.999
92	0.285	0.883	0.825	2.35	0.958	0.906	0.999
94	0.333	1.066	1.123	2.20	0.797	0.591	0.999
96	0.370	1.350	1.317	2.52	0.545	1.267	0.999
100	0.400	2.333	2.160	2.6	0.401	0.0933	0.999
101	0.434	2.667	2.459	2.68	0.354	0.0622	0.999
102	0.467	2.816	2.752	2.62	0.314	0.0484	0.999
103	0.418	4.349	4.185	2.47	0.206	0.466	0.999
104	0.500	5.249	4.987	2.84	0.185	0.333	0.999
105	0.534	5.900	5.710	2.19	0.166	0.534	0.999
108	0.533	5.733	5.225	2.55	0.166	0.533	0.999
110	0.667	8.682	7.845	2.43	0.116	0.667	0.999

The degree of crystallinity (x_c) obtained at the end of the isothermal crystallization process for all samples was calculated as:

$$x_c = \frac{\Delta H_{iso}}{\Delta H^0} * 100$$

The ΔH_{iso} value is the saturation enthalpy obtained during the isothermal crystallization process at the chosen T_c. The ΔH^0 is the enthalpy corresponding to a 100% crystalline PHB (146 J/g, [1]).

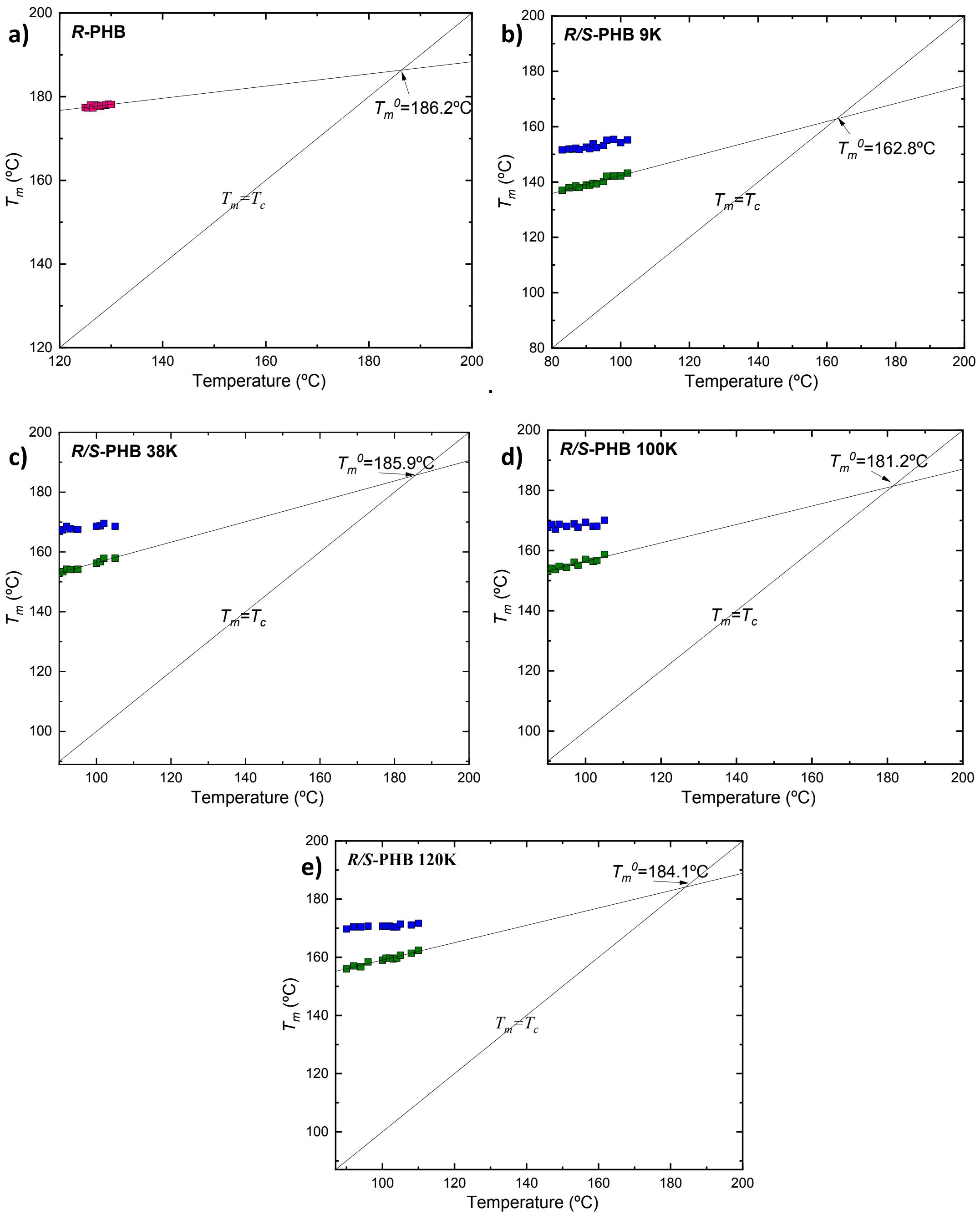

Figure S8 Hoffman-Weeks plots for *R* PHB (a), *R/S* PHB 8K (b), 38K (c), 100K (d), and 120K (e).

Table S14 Equilibrium melting temperature values reported in the literature.

	$\boldsymbol{T_m^0}$ **(ºC)**
Barham et al. PHB[1]	195.0
Greco et al.PHB [6]	196.0
Avella et al.[7]	194.0
Paglia et al.PHB [8]	187.9

REFERENCES

(1) Barham, P. J.; Keller, A.; Otun, E. L.; Holmes, P. A. Crystallization and Morphology of a Bacterial Thermoplastic: Poly-3-Hydroxybutyrate. *J. Mater. Sci. 1984 199* **1984**, *19* (9), 2781–2794. https://doi.org/10.1007/BF01026954.

(2) Hoffman, J. D.; Davis, G. T.; Lauritzen, J. I. The Rate of Crystallization of Linear Polymers with Chain Folding. In *Treatise on solid state chemistry*; Springer, 1976; pp 497–614. https://doi.org/1 O.l 007/978-1-4684-2664-9.

(3) Pitt, C. G.; Chasalow, F. I.; Hibionada, Y. M.; Klimas, D. M.; Schindler, A. Aliphatic Polyesters. I. The Degradation of Poly (E-caprolactone) in Vivo. *J. Appl. Polym. Sci.* **1981**, *26* (11), 3779–3787. https://doi.org/https://doi.org/10.1002/app.1981.070261124.

(4) Mark, J. E. *Physical Properties of Polymers Handbook*; Springer, 2007; Vol. 1076.

(5) Pérez-Camargo, R. A.; Liu, G.; Wang, D.; Müller, A. J. Experimental and Data Fitting Guidelines for the Determination of Polymer Crystallization Kinetics. *Chinese J. Polym. Sci* **2022**. https://doi.org/https://doi.org/10.1007/s10118-022-2724-2.

(6) Greco, P.; Martuscelli, E. Crystallization and Thermal Behaviour of Poly(d(—)-3-Hydroxybutyrate)-Based Blends. *Polymer (Guildf).* **1989**, *30* (8), 1475–1483. https://doi.org/10.1016/0032-3861(89)90219-X.

(7) Avella, M.; Martuscelli, E.; Greco, P. Crystallization Behaviour of Poly(Ethylene Oxide) from Poly(3-Hydroxybutyrate)/Poly(Ethylene Oxide) Blends: Phase Structuring, Morphology and Thermal Behaviour. *Polymer (Guildf).* **1991**, *32* (9), 1647–1653. https://doi.org/10.1016/0032-3861(91)90401-4.

(8) Paglia, E. D.; Beltrame, P. L.; Canetti, M.; Seves, A.; Marcandalli, B.; Martuscelli, E. Crystallization and Thermal Behaviour of Poly (D (−) 3-Hydroxybutyrate)/Poly (Epichlorohydrin) Blends. *Polymer (Guildf).* **1993**, *34* (5), 996–1001. https://doi.org/https://doi.org/10.1016/0032-3861(93)90220-5.
